# Genetic Inhibition of CaMKII in Dorsal Striatal Medium Spiny Neurons Reduces Functional Excitatory Synapses and Enhances Intrinsic Excitability

**DOI:** 10.1371/journal.pone.0045323

**Published:** 2012-09-21

**Authors:** Jason R. Klug, Brian N. Mathur, Thomas L. Kash, Hui-Dong Wang, Robert T. Matthews, A. J. Robison, Mark E. Anderson, Ariel Y. Deutch, David M. Lovinger, Roger J. Colbran, Danny G. Winder

**Affiliations:** 1 Department of Molecular Physiology and Biophysics, Vanderbilt University School of Medicine, Nashville, Tennessee, United States of America; 2 Center for Molecular Neuroscience, Vanderbilt University School of Medicine, Nashville, Tennessee, United States of America; 3 J.F. Kennedy Center for Research on Human Development, Vanderbilt University School of Medicine, Nashville, Tennessee, United States of America; 4 Department of Psychiatry, Vanderbilt University Medical Center, Nashville, Tennessee, United States of America; 5 Department of Pharmacology, Vanderbilt University Medical Center, Nashville, Tennessee, United States of America; 6 Laboratory for Integrative Neuroscience, National Institute on Alcohol Abuse and Alcoholism, National Institutes of Health, Rockville, Maryland, United States of America; 7 Departments of Internal Medicine and Molecular Physiology and Biophysics, University of Iowa, Iowa City, Iowa, United States of America; Northwestern University, United States of America

## Abstract

Ca^2+^/calmodulin-dependent protein kinase II (CaMKII) is abundant in striatal medium spiny neurons (MSNs). CaMKII is dynamically regulated by changes in dopamine signaling, as occurs in Parkinson's disease as well as addiction. Although CaMKII has been extensively studied in the hippocampus where it regulates excitatory synaptic transmission, relatively little is known about how it modulates neuronal function in the striatum. Therefore, we examined the impact of selectively overexpressing an EGFP-fused CaMKII inhibitory peptide (EAC3I) in striatal medium spiny neurons (MSNs) using a novel transgenic mouse model. EAC3I-expressing cells exhibited markedly decreased excitatory transmission, indicated by a decrease in the frequency of spontaneous excitatory postsynaptic currents (sEPSCs). This decrease was not accompanied by changes in the probability of release, levels of glutamate at the synapse, or changes in dendritic spine density. CaMKII regulation of the AMPA receptor subunit GluA1 is a major means by which the kinase regulates neuronal function in the hippocampus. We found that the decrease in striatal excitatory transmission seen in the EAC3I mice is mimicked by deletion of GluA1. Further, while CaMKII inhibition decreased excitatory transmission onto MSNs, it increased their intrinsic excitability. These data suggest that CaMKII plays a critical role in setting the excitability rheostat of striatal MSNs by coordinating excitatory synaptic drive and the resulting depolarization response.

## Introduction

The striatum is the major input nucleus of the basal ganglia [1]. Dysfunction in this region is associated with drug addiction, Parkinson's disease and other disorders [2], [3], [4], [5], [6], [7], [8]. The striatum is primarily composed of projection GABAergic medium spiny neurons (MSNs) that integrate glutamatergic excitatory transmission with modulatory dopaminergic transmission. Since MSN firing is thought to be driven primarily by excitatory drive, understanding the basic mechanisms of glutamatergic transmission onto MSNs is necessary to understand how the striatum functions in health and disease.

Calcium-calmodulin-dependent kinase II (CaMKII) is a Ser/Thr kinase that is highly expressed in the striatum, constituting ∼0.7% of total striatal protein [9]. CaMKII assembles into dodecameric complexes that in the striatum predominantly contain CaMKIIα and CaMKIIβ isoforms [10]. As a major constituent of the postsynaptic density (PSD) in the dorsal striatum [11] as well as other forebrain regions [12], [13], CaMKII is activated by N-methyl-D-aspartate-receptor (NMDAR)-mediated calcium influx [14], [15], [16]. CaMKII is a key modulator of hippocampal and cortical pyramidal cell glutamate synapse function [17], [18], [19]. CaMKII can phosphorylate many downstream substrates including the ionotropic glutamate receptors NMDARs and α-amino-3-hydroxy-5-methyl-4-isoxazolepropionic acid receptors (AMPARs) [20], [21], [22], [23], [24], [25]. Indeed, in hippocampal pyramidal cells, CaMKII activation enhances synaptic trafficking of AMPARs and channel function [26], [27], [28], [29]. In addition, a constitutively active form of CaMKII can decrease intrinsic excitability of hippocampal neurons as well as MSNs in the nucleus accumbens shell [30], [31]. While much is known about the role of CaMKII at glutamate synapses on glutamatergic projection neurons such as hippocampal and cortical pyramidal neurons, relatively little is known for GABAergic cells. Indeed, little CaMKII is expressed in GABAergic interneurons [32], [33], [34], making GABAergic projection cells such as MSNs, which are highly enriched in CaMKII, unique targets for studying the role of CaMKII in synaptic transmission and intrinsic excitability.

Previous studies have implicated striatal CaMKII in Parkinson's disease (PD) and addiction. CaMKII is hyperactivated after striatal dopamine depletion, and CaMKII inhibition rescued striatal synaptic plasticity and motor deficits found in animal models of Parkinson's disease [35]. Striatal CaMKII regulates motivational effects of reward cues on goal-directed behaviors [36] as well as curbing D1R-mediated cocaine hyperlocomotion [37] and modulating excitability following chronic cocaine administration[31]. Thus, a better understanding of CaMKII's role in striatal glutamatergic synaptic transmission may suggest new approaches to treat PD and addiction.

In addition to its postsynaptic roles, CaMKII modulates a variety of presynaptic functions, including trafficking of synaptic vesicles [38], [39], [40], [41], [42], P/Q type calcium channels [43], [44], [45], voltage-gated sodium channels [46], [47], catecholamine synthesis [48], [49] and dopamine transporter function [50], [51]. Thus, an investigation of the role of CaMKII within striatal MSNs requires a cell-specific approach. To accomplish this, we generated a transgenic mouse line that expresses a CaMKII inhibitory peptide selectively within dorsal striatal MSNs. Using this line, we found that CaMKII inhibition in dorsal striatal MSNs leads to a loss of functional glutamatergic synapses and an increase in intrinsic excitability. These findings shed light on the neural mechanisms underlying the development of striatal neural circuits, learning and memory, and motor behavior.

## Materials and Methods

### Generation of EAC3I-4 transgenic mice

For generation of double transgenic EAC3I-4 X tTA animals, heterozygous transgenic mice carrying the tTA gene driven by an alpha CaMKII promoter fragment were bred to heterozygous mice carrying the EAC3I transgene fused to EGFP driven by the tetO promoter. The CaMKIIα-tTA mice were obtained from Dr. Eric Kandel's lab and maintained at Vanderbilt University. The autocamtide-3 derived inhibitory peptide (EAC3I) sequence (KKALHRQEAVDAL) mimics the autoinhibitory region of the CaMKII regulatory domain (residues 278–290) and acts by competitively binding to the catalytic site. In *in vitro* biochemical assays AC3-I blocks the phosphorylation of an autocamtide-2 substrate by purified rat CaM kinase with an IC_50_ of 3 µM [52], [53], with a ≥100-fold reduced potency toward protein kinase C, CaM kinase I or CaM kinase IV [52], [54], [55]. EAC3-I is made up of the AC3-I peptide fused N-terminal to enhanced green fluorescent protein (EGFP) to stabilize and mark cellular and tissue distribution. In a previous study, EAC3I was transgenically expressed in the heart and total CaMKII activity in extracts was reduced by ≈40% [56]. This level of inhibition is likely to be a substantial underestimate of *in vivo* inhibition, because proteins were diluted upon homogenization due to mosaic transgene expression.

We quantified the level of mosaicism in our EAC3-I mouse by staining with a NeuN antibody (1∶1000, Millipore) to label all neurons (See supplemental for detailed immunohistochemical labeling). Manual counts of the number of EGFP positive neurons versus total number of NeuN stained neurons in z-stacks in the dorsal lateral striatum were made in Metamorph (Molecular Devices; Sunny Vale, CA), providing an estimate of the percent of cells expressing the transgene. TetO-linked transgene expression is controlled using mouse chow containing 200 mg/kg Doxycycline (DOX) (Bio-Serv; Frenchtown, NJ). For DOX rescue experiments pregnant dams were fed DOX and weaned pups continued with the same food. At 6 weeks DOX was removed and the transgene was allowed to be expressed for 4–5 weeks. All DOX recordings were made between 10–11 weeks. All mice had been inbred onto a C57BL/6 background for more than seven generations. GluA1 knockout mice (Andrew Holmes Lab) and wildtype littermates 8–16 weeks of age were utilized [57].

### Brain Slice Preparation

All procedures were performed in accordance with the Vanderbilt University Institutional Animal Care and Use Committee and National Institutes of Health Guide for Care and Use of Laboratory Animals and approved by the Vanderbilt and NIAAA Animal Care and Use Committee. Male and female EAC3I-4 transgenic mice or littermate controls (9–13 weeks or 3–4 weeks animals when indicated) were decapitated under anesthesia (Isoflurane). The brains were quickly removed and placed in ice-cold sucrose-artificial cerebrospinal fluid (ACSF): (in mM) 194 sucrose, 20 NaCl, 4.4 KCl, 2 CaCl_2_, 1 MgCl_2_, 1.2 NaH_2_PO4, 10.0 glucose, and 26.0 NaHCO_3_ saturated with 95% O_2_/5% CO_2_. Hemisected coronal slices 300 µm in thickness were prepared using a Tissue Slicer (Leica). Slices containing dorsal lateral striatum were collected rostral to the crossing of the anterior commissure (Bregma 1.10–0.2 mm) (Franklin and Paxinos 1997). Slices were then stored in a heated (approximately 28°C), oxygenated (95% O_2_-5% CO_2_) holding chamber containing ‘normal’ ACSF [ACSF: (in mM) 124 NaCl, 4.4 KCl, 2 CaCl_2_, 1.2 MgSO_4_, 1 NaH_2_PO_4_, 10.0 glucose, and 26.0 NaHCO_3_] for 1 hour and then transferred to a submersion-type recording chamber (Warner Instruments) where they were superfused with heated (28°C) oxygenated ACSF at a rate of about 2–3 ml/min. Preparation of GluA1KO animals and controls brain slices used very similar methodology except the high sucrose ACSF contained (in mM): 194 sucrose, 30 NaCl, 4.5 KCl, 1 MgCl_2_, 26 NaHCO_3_, 1.2 NaH_2_PO_4_, and 10 glucose and 250 µm thick brain slices were placed in 30°C oxygenated ACSF containing (in mM): 124 NaCl, 4.5 KCl, 2 CaCl_2_, 1 MgCl_2_, 26 NaHCO_3_, 1.2 NaH_2_PO_4_, and 10 glucose for 30 minutes followed by 30 minutes at room temperature before moving hemisections to the recording chamber.

### Whole-Cell Voltage Clamp Recordings

MSNs of the dorsal lateral striatum were directly visualized with infrared video microscopy (Olympus BX51WI with QImaging Rolera-XP Camera). Only highly expressing EGFP-containing MSNs were selected for study and compared to neighboring MSNs visually devoid of EGFP expression. Recording electrodes (3–6 MΩ) were pulled on Flaming-Brown Micropipette Puller (Sutter Instruments) using thin-walled borosilicate glass capillaries (WPI). EPSCs were evoked by local fiber stimulation with bipolar nichrome electrodes. Stimulating electrodes were placed on the border of the corpus callosum and dorsal lateral striatum 100–300 µm dorsal to the recorded neuron, and electrical stimulation (5–20 V with 100–150 µs duration, Grass Instruments) was applied at 0.05 Hz unless otherwise noted. This location most likely stimulates both cortical and thalamic glutamatergic axons onto MSNs. Recording electrodes (3–6 MΩ) were filled with (in mM) Cs+ gluconate (117), HEPES (20), EGTA (0.4), TEA (5), MgCl_2_ (2), ATP (4), GTP (0.3) pH 7.35, 285–290 mOsm. Series resistance averaging 16 MΩ (ranging 8–30 MΩ) was monitored and experiments with changes greater than 20% were omitted. AMPAR EPSCs and sEPSCs were isolated by adding 25 µM picrotoxin and recording at a holding potential of −70 mV in normal ACSF. To isolate mEPSCs 1 uM TTX was added in addition to sEPSCs recording conditions. In all experiments a time period of at least 5 minutes post break in was allowed for internal solution exchange and stabilization of membrane properties. GluA1KO sEPSC recordings were conducted similarly except recording ACSF contained 50 µM picrotoxin and internal solution contained (in mM) 120 CsMeSO_3_, 5 NaCl, 10 TEA-Cl, 10 HEPES, 5 QX-314, 1.1 EGTA, 0.3 Na-GTP, and 4 Mg-ATP, 295–300 mOsm. GluA1 WT and KO littermate mice were 8–16 weeks of age at time of recording. In PPR experiments evoked 100–200 pA responses were elicited with the interstimulus interval set at 40 ms, 50 ms and 60 ms. For MK-801 experiments NMDA currents were pharmacologically isolated (25 µM picrotoxin, 10 µM NBQX) and held at +40 mV while a stable ten minute baseline (0.05 Hz) was acquired. After a stable baseline was acquired the stimulator was switched off and 10 µM MK-801 was washed on for seven minutes. Following the wash-in period the stimulator was turned on (0.1 Hz) and the time constant for decay of the integral of the NMDA current was calculated using nonlinear regression one-phase decay. For baclofen modulation of PPR, 20 sweeps of approximately 200 pA EPSCs (0.05 Hz) were collected to establish a baseline. Following a wash-in of 10 uM baclofen for 8 minutes, an additional 20 sweeps were collected. Additional 20 sweeps were taken at 10 minutes and 20 minutes post washout. CV was calculated by dividing the SD of the amplitude of the evoked EPSCs by the mean. For rectification experiments, evoked AMPAR-mediated EPSCs were isolated in 25 µm picrotoxin and 100 µm DL-APV containing aCSF while the voltage was stepped from −70 mV to +40 mV in 10 mV steps. 0.1 mM spermine was included in the standard cesium internal solution to avoid dialysis of endogenous polyamines. The rectification index (RI) was calculated as the ratio of the amplitude of AMPAR-mediated currents evoked at −70 mV over +40 mV. All signals were acquired via a Multiclamp 700B amplifier (Axon Instruments), digitized at 10 kHz, filtered at 2 kHz and analyzed via pClamp 10.2 software (Axon Instruments). Holding current and series resistance were all monitored continuously throughout the duration of experiments. Experiments in which changes in series resistance were greater than 20% were not included in the data analysis.

Statistical analyses were performed using Graphpad Prism 5.04. Two-tailed unpaired Student's t-test (t) were used unless variance differed significantly (Bartlett's test for equal variances) then non-parametric Mann-Whitney (U) tests were used. One or Two-way analysis of variance (ANOVA) (F) were used when indicated with Neuman-Keuls Multiple Comparison post hoc test. Non-parametric Kruskal-Wallis tests (H) were used with Dunn's Multiple Comparison post hoc test when variances differed significantly (Bartlett's test for equal variances). All values given are presented as average ± SEM. Cumulative probability plots were analyzed with Kolmogorov-Smirnov (KS) test.

### Whole cell current clamp recordings

Slices were prepared as before, but perfused with ACSF containing (in mM): NaCl (124), NaH_2_PO_4_ (1.25), KCl (2.5), CaCl_2_ (2.5), MgSO_4_ (2), NaHCO_3_ (26), Glucose (11) pH = 7.35, 300–305 mOsm. Recording electrodes were filled with in (mM): K+ gluconate (120), NaCl (4), HEPES (10), Mg-ATP (4), Na-GTP (0.3), KCl (20), Na+ Phosphocreatine (10) pH = 7.3, 285–290 mOsm. MSNs were identified by their intrinsic membrane properties (i.e. resting membrane typically more negative than −80 mV, inward and outward rectification in response to somatic positive and negative current injections, and a long depolarizing ramp to delayed first spike discharge [58]. Recordings were rejected if the initial Vm was more positive than −75 mV. Resting membrane potential or zero current potential was determined right upon break-in. Spontaneous excitatory postsynaptic potentials after 5 minutes post break in were recorded for 4 minutes with the cell dynamically current clamped at −85 mV. Resting membrane potential was monitored and current was injected to maintain the resting potential at −85 mV. For current-voltage (IV) relationships positive or negative current injections were given in 20 pA steps until the cell reached threshold and fired a single AP. Five additional current injections steps (20 pA each) were given above threshold. Input resistance was monitored throughout the experiment and the cell was rejected if the input resistance changed by more than 20%. Healthy cells showed APs that crossed +30 mV and stable resting membrane potentials.

### Lucifer yellow intracellular fills, confocal imaging and measurement of dendritic structure

Transgenic EAC3I mice (3–4 months) were perfused with 10 ml room temperature phosphate buffered saline (PBS) followed by 100 ml of 4% paraformaldehyde solution delivered over 10–20 minutes. Brains were post-fixed in ice cold paraformaldehyde solution for an additional hour before 200 µm thick coronal sections of the precommissural striatum were prepared on a vibrating microtome (Leica; Buffalo Grove, IL). MSN cell bodies in dorsolateral striatum were visualized at 40×. Randomly selected EAC3I-expressing (EGFP-positive) MSNs and non-fluorescent (EGFP-negative) MSNs in the dorsal lateral striatum were iontophoretically filled with an 8% solution of Lucifer yellow (LY; Sigma-Aldrich, in 50 mM Tris-HCl, pH 7.4) using hyperpolarizing current (3–5 nA for 8–10 minutes). Slices were fixed in 4% paraformaldehyde/PBS overnight at 4°C and then coverslipped using Prolong Gold mounting solution (Invitrogen; Grand Island, NY).

Digital images of MSN dendritic segments located 80–100 µm distal to the cell soma were captured using a Zeiss LSM 710 confocal microscope with ×63 oil immersion objective and ×2.5 digital zoom. Spine density of three to four dendritic segments emanating from different primary dendrites was averaged to yield a mean spine density value per MSN. A total of 17 EAC3I-negative MSNs and 16 EAC3I-positive MSNs were analyzed; these were obtained from four different mice of each genotype. After the confocal images were coded by someone not involved in the study, another person unaware of the animal or genotype of the MSN being examined used Imaris (Version 5.5; Bitplane) to quantify dendritic spine density. A three-dimensional perspective in “surpass” mode of the software package was generated and images were processed with background subtraction thresholding and smoothed with a Gaussian filter. Dendritic segments were modeled with the largest and smallest diameters set at 2 µm and 1 µm, respectively. Florescence in each dendritic segment was thresholded manually to capture all dendritic spines. The minimum terminal point spine diameter was set at 0.143 µm and the florescence contrast threshold was set at 1. Identified spines were counted and marked in 3D on a rotating version of the image. Finally, each structure identified as a spine by the Imaris software was visually verified. In order to determine total dendritic length and generate a Sholl analysis (number of dendritic branches intersecting circular rings drawn around the soma every 20 µm distal to the soma), we used Neurolucida Explorer (MicroBrightField; Willington, VT).

### Pharmacology

Picrotoxin, (+)Mk-801 maleate ((5S,10R)-(+)-5-Methyl-10,11-dihydro-5H-dibenzo[a,dicyclohepten-5,10-imine maleate), NBQX (2,3-Dioxo-6-nitro-1,2,3,4-tetrahydrobenzo[f]quinoxaline −7-sulfonamide), (R)-Baclofen ((*R*)-4-Amino-3-(4-chlorophenyl)butanoic acid), and DL-APV (DL-2-Amino-5-phosphonopentanoic acid) were purchased from Tocris (Ellisville, Missouri). DMSO (0.05%) (Sigma-Aldrich) was used as a vehicle for picrotoxin.

## Results

### Characterization and *in vivo* localization/expression of CaMKII inhibitory (EAC3I) peptide

**Figure 1:**
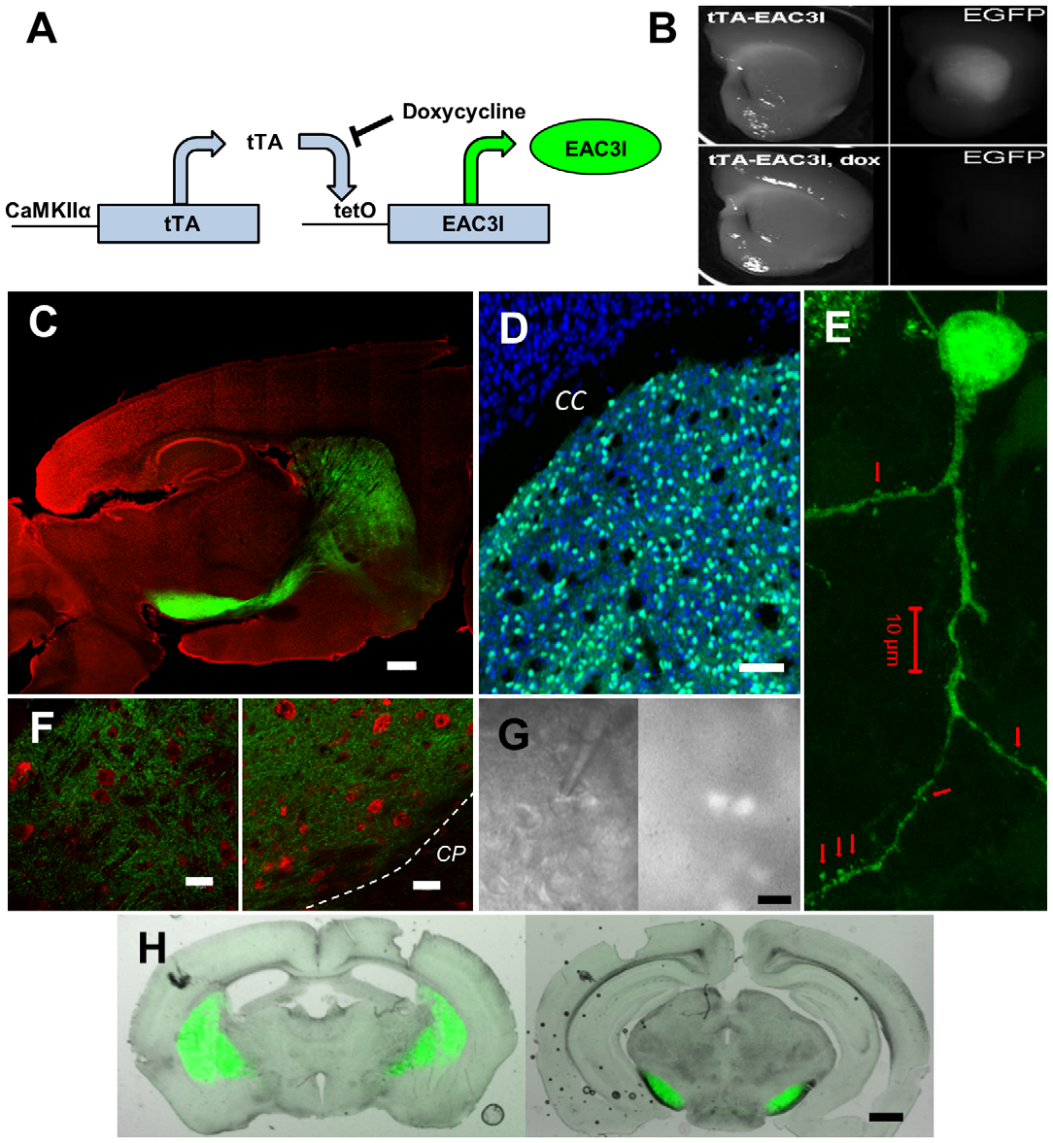
Characterization and *in vivo* localization/expression of CaMKII inhibitory (EAC3I) peptide in EAC3I mice. (A) Schematic of breeding strategy for production of EAC3I mice. CaMKIIalpha promoter drives expression of tTA which binds to the tetO promoter and drives expression of EAC3I peptide fused to EGFP. (B) (Top) Brain slice of EAC3I under brightfield (left) and EGFP epifluorescence (right). (Bottom) Brain slice of EAC3I as above following 3 weeks of doxycycline feeding (200 mg/kg). Note lack of EAC3I-EGFP expression. (C) Sagittal section of EAC3I mouse brain showing restricted endogenous EAC3I-EGFP expression (green) and nissl stain (red, neuronal marker). Scale bar 500 µm. (D) Coronal image of mosaic expression of endogenous EAC3I-EGFP expression (green) in dorsal lateral striatum with a NeuN stain (blue, neuronal marker). Note little to no expression of EAC3I peptide in cortex. CC = corpus callosum, Scale bar 100 µm. (E) 63× image of unstained EAC3I-EGFP expressing MSN (green = endogenous EGFP signal). Note expression in soma, dendrites and dendritic spines (arrows). Scale bar 10 µm. (F) Images of globus pallidus (GP) (left) and substantia nigra pars reticulata (SNR) (right) showing MSN axon terminals (green) and nissl stain (red). GP and SNR cell somas (red, nissl stain) are devoid of EGFP signal. CP = cerebral peduncle. Scale bars 20 µm. (G) (left) DIC image of patch pipette on a MSN in whole cell mode, (right) epifluorescence image of left panel confirming EAC3I-EGFP expression. Scale bar 20 µm. (H) Overlaid coronal images showing EAC3I-EGFP MSN axon terminal field expression in globus pallidus (left) and substania nigra pars reticulata (right) confirming indirect and direct pathway expression, respectively. Scale bar 0.5 mm.

To determine the role CaMKII plays in modulating glutamatergic transmission onto MSNs in the dorsal lateral striatum, we generated a transgenic mouse model with striatally-enriched expression of a CaMKII inhibitory peptide fused to enhanced green fluorescent protein (EGFP) referred to hereafter as EAC3I [52]. The EAC3I peptide inhibits all isoforms of CaMKII, as well as both calcium-dependent and independent forms of the kinase, and the fusion with EGFP allows for visualization of the regional and cellular distribution of the transgenically expressed protein. EAC3I was previously utilized in another transgenic line to examine the role of CaMKII in the heart [56]. To spatially and temporally regulate EAC3I expression, the tetracycline transactivator (tTA) is driven by a CaMKIIα promoter fragment with the tTA gene product driving expression of EAC3I (Figure 1A). Constitutive expression of the EAC3I transgene has no overt effect on viability or on gross brain morphology, and is silenced by including doxycycline (DOX, 200 mg/kg) in the animals' chow for two weeks (Figure 1B). The alpha CaMKII promoter normally restricts transgene expression to the forebrain, but this founder line exhibits enrichment of EAC3I expression in the MSNs of the dorsal striatum (Figure 1C, 1D), presumably due to integration site-dependent effects. Little to no expression of the inhibitor was seen in cortex and thalamus (Figure 1C, 1D). The EAC3I peptide is expressed in a mosaic pattern throughout the dorsal striatum, with 34±4% of neurons containing the inhibitory peptide (Figure 1D). The mosaicism observed is common in transgenic animals and different founder lines utilizing the alpha CaMKII promoter fragment to drive transgene expression show differing expression patterns throughout the forebrain [59], [60]. However, we observed no overlap of signal between EGFP positive neurons and striatal cholinergic and GABAergic interneuron markers such as ChaT, parvalbumin, NPY, and calretinin (Figure S1). At higher magnification the EAC3I inhibitory peptide was observed in the MSN cell soma, dendrites and dendritic spines (Figure 1E). Dense expression of EAC3I was detected in both the globus pallidus and in the substantia nigra pars reticulata (Figure 1C, 1F, 1H), but higher magnification images revealed the staining was localized to axons (Figure 1F), demonstrating that both indirect and direct pathway MSNs contain the inhibitory peptide (Figure 1C, 1F, 1H).

### Effects of CaMKII inhibition on MSN glutamatergic inputs in dorsal lateral striatum

We initially recorded sEPSCs from MSNs using whole-cell voltage clamp in the presence of picrotoxin to isolate excitatory transmission. In this and subsequent electrophysiological analyses, we compared three control groups of MSNs with one experimental group: Control 1) MSNs from tTA-/tetO-EAC3I- mice (Wt), Control 2) MSNs from tTA+/tetO-EAC3I- mice (tTA), Control 3) EGFP negative MSNs from tTA+/tetO-EAC3I+ mice (NON EGFP) and Experimental 4) EGFP positive MSNs from tTA+/tetO-EAC3I+ mice (referred to as EAC3I MSNs (EGFP)).

**Figure 2:**
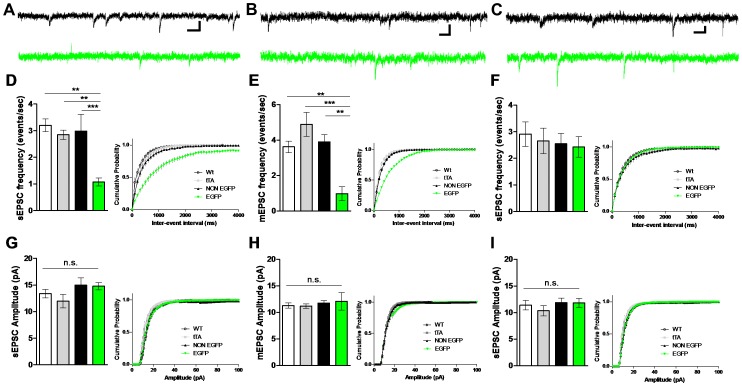
*In vivo* expression of EAC3I decreases s/mEPSC frequency in dorsal lateral striatum MSNs. (A) Representative sEPSC traces for NON EGFP (black) and EGFP (CaMKII-inhibited, green). Scale bars 50 ms and 10 pA. (B) Representative mEPSC traces for NON EGFP (black) and EGFP (CaMKII-inhibited, green). Scale bars 50 ms and 10 pA. (C) Representative sEPSC traces for NON EGFP (black) and EGFP (CaMKII-inhibited, green) MSNs from animals that were fed DOX from birth to six weeks and then removed to allow EAC3I transgene expression. Recordings were made 4–5 weeks following DOX removal at a similar age to previous. The scale bars are 50 ms and 10 pA. (D) (Left) Average sEPSC frequencies from EAC3I-containing MSNs compared to controls, (right) Cumulative probability distributions of sEPSC inter-event intervals (Wt: n = 7, p = 0.0001; tTA: n = 5, p = 0.0001; Non EGFP: n = 9, p = 0.0009; versus EGFP: n = 13). (E) (left) Average mEPSC frequency from EAC3I MSNs compared to controls, (right) cumulative probability distributions of mEPSC frequency (Wt: n = 5, p = 0.0022; tTA: n = 6, p<0.0001; Non EGFP: n = 6, p = 0.0019; versus EGFP: n = 6). (F) (Left) Average sEPSC frequencies from EAC3I-containing MSNs versus controls, (right) cumulative probability distributions of sEPSC inter-event intervals (Wt: n = 8, p = 0.99; tTA: n = 7, p = 0.99; Non EGFP: n = 8, p = 0.99; versus EGFP: n = 10). (G) (left) Average sEPSC amplitudes, (right) cumulative probability distributions of sEPSC amplitude (Wt: n = 7, p = 0.91; tTA: n = 5, p = 0.26; Non EGFP: n = 9, p = 0.99; versus EGFP: n = 13). (H) (left) Average mEPSC amplitude, (right) cumulative probability distributions of mEPSC amplitude (Wt: n = 5, p = 0.90; tTA: n = 6, p = 0.71; Non EGFP: n = 6, p = 0.96; versus EGFP: n = 6). (I) (left) Average sEPSC amplitudes, (right) cumulative probability distributions of sEPSC amplitude from animals that were fed DOX from birth to six weeks and then removed to allow EAC3I transgene expression (Wt: n = 8, p = 0.99; tTA: n = 7, p = 0.34; Non EGFP: n = 8, p = 0.99; versus EGFP: n = 10). * P<0.05; ** P<0.01; *** *P<0.001;* error bars represent SEM, N.S. = not significant. Note: All neurons in panels A-I are held at −70 mV during recordings.

In adult mice no differences in sEPSC amplitudes were observed between MSNs from the four groups described above (in pA, Wt 13.39±0.81; tTA 11.98±1.27; NON EGFP 14.97±1.38; EGFP 14.78±0.67) [F (3, 33) = 1.46; p = NS; Figure 2A, 2G]. However, sEPSC frequency was markedly reduced in EAC3I MSNs relative to all control MSN groups (in Hz, Wt 3.20±0.24; tTA 2.85±0.17; NON EGFP 2.99±0.63; EGFP 1.07±0.15) [H (3, 33) = 19.85; p = 0.0002]; Figure 2A, 2D]. Similar results were observed in sEPSC frequency in 3–4 week old mice (in Hz, NON EGFP 3.94±1.05; EGFP 1.08±0.23) [U (9) = 3.000; p = 0.0341; data not shown]. Additionally, we did not observe any differences in AMPAR-mediated current voltage relationship between EAC3-I containing and lacking MSNs (Rectification Index (RI) = −70 mV/+40 mV, NON EGFP 1.97±0.18; EGFP 1.75±0.24) [t (8) = 0.575; p = 0.58; data not shown]. To determine if the decrease in sEPSC frequency was due to a change in the intrinsic electrical activity in the slice, we also examined activity-independent miniature EPSCs (mEPSCs) in the presence of TTX. Similar to the results with sEPSCs, mEPSC frequency was also reduced in EAC3I MSNs (in Hz, Wt 3.62±0.32; tTA 4.88±0.67; NON EGFP 3.89±0.41; EGFP 0.97±0.40) [F (3, 22) = 9.688; p = 0.0008]; Figure 2B, 2E], suggesting that this effect is independent of presynaptic excitability.

The use of the tTA expression system provides DOX-dependent control of EAC3I expression (Figure 1B). We suppressed expression of the transgene by supplementation of food with DOX (200 mg/kg) for both the dam and her litter until 6 weeks of age, and then recorded from EAC3I-expressing MSNs 4 weeks after removal of DOX. Under these conditions, the global expression of EAC3I was markedly lower than in non-DOX exposed mice, as has been previously noted with the tTA expression system [61]. Notably, sEPSC frequency in the EAC3I MSNs was not significantly different from controls (in Hz, Wt 2.91±0.46; tTA 2.65±0.48; NON EGFP 2.55±0.39; EGFP 2.42±0.39) [F (3, 32) = 0.2434; p = NS]; Figure 2C, 2F]. These data indicate that the sEPSC frequency phenotype observed in no-DOX EAC3I MSNs is not likely due to an insertion site artifact.

### MSN CaMKII inhibition reduces excitatory transmission independently of changes in release probability

**Figure 3:**
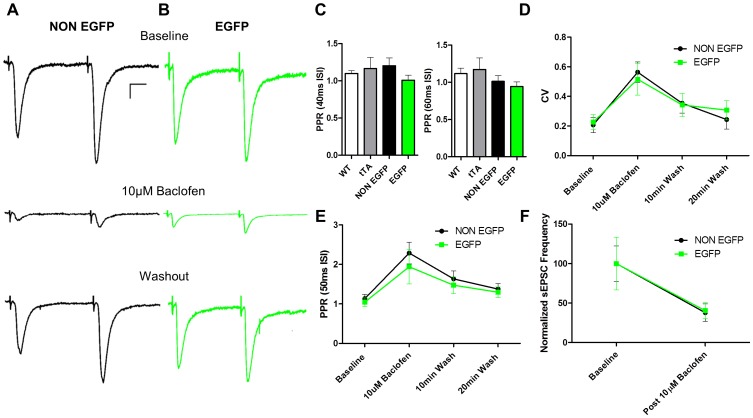
Dorsal lateral striatum MSN CaMKII inhibition reduces excitatory transmission independently of changes in release probability. (A) (Upper trace, black) NON EGFP (EAC3I-lacking) MSN PPR (50 ms ISI) example trace baseline (average of 20 sweeps, 0.05 Hz). (Middle trace, black) EAC3I-lacking MSN PPR (50 ms ISI) example trace post 10 µM Baclofen wash in (average of 20 sweeps, 0.05 Hz). (Bottom trace, black) EAC3I-lacking MSN PPR (50 ms ISI) example trace 20 minutes post wash out of drug (average of 50 sweeps, 0.05 Hz). (B) Same as in (A), but for an EGFP (EAC3I-containing) MSN. (C) Average PPR recorded by paired-pulse stimulation eliciting EPSCs with two different interstimulus intervals (40 and 60 ms) for EAC3I-containing MSNs versus all controls. (Wt: n = 9; tTA: n = 6; NON EGFP: n = 9; EGFP: n = 10; 40 ISI p = 0.38, 60 ISI p = 0.27). (D) Baclofen increases the CV of EPSCs. Coefficient of variation (CV = SD/Mean) change of EPSCs from before, during and after application of baclofen for EAC3I-containing and EAC3I-lacking MSNs (NON EGFP: n = 6; EGFP: n = 5; p = 0.95). (E) Baclofen increases the PPR. PPR (50 ms ISI) for EAC3I-containing and EAC3I-lacking MSNs before, during and after application of baclofen (NON EGFP: n = 6; EGFP: n = 5; p = 0.48). (F) Baclofen decreases sEPSC frequency. Plot of normalized sEPSC frequency post 10 µM Baclofen compared to baseline for EAC3I-containing and EAC3I-lacking MSNs (NON EGFP: n = 6; EGFP: n = 5; p = 0.96). Note: All MSNs held at −70 mV during recordings.

The canonical interpretation of a reduction in s/mEPSC frequency is via a reduction in the probability of glutamate release or the number of release sites/number of synapses. In order to better understand the mechanism(s) underlying the reduction in s/mEPSC frequency in EAC3I MSNs we first examined paired-pulse ratios (PPR) of evoked EPSCs, a measurement that inversely correlates with neurotransmitter release probability [62]. The PPR of evoked EPSCs on control MSNs did not differ from that observed on EAC3I MSNs [PPR 40 ms ISI: (Wt 1.10±0.03; tTA 1.16±0.15; NON EGFP 1.20±0.10; EGFP 1.01±0.07) H (3, 33) = 2.436; p = NS; PPR 60 ms ISI: (Wt 1.12±0.07; tTA 1.17±0.16; NON EGFP 1.01±0.07; EGFP 0.94±0.06) F (3, 33) = 1.377; p = NS; Figure 3A, 3B, 3C]. To show that we could predictably manipulate PPR we used the GABA_B_R agonist baclofen (10 µM). Baclofen acts presynaptically to reduce the probability of release and therefore increase PPR at a number of CNS synapses [62], [63]. Baclofen increased the PPR in EAC3I MSNs to a similar extent as in control MSNs suggesting that release probability was modifiable in the CaMKII-inhibited cells and not at a floor (NON EGFP baseline 1.13±0.11, 10 µM baclofen 2.29±0.28, 20 min washout 1.38±0.14; EGFP baseline 1.04±0.11, 10 µM baclofen 1.94±0.43, 20 min washout 1.30±0.14) [F (1, 9) = 0.5403; p = NS; Figure 3E]. Similar results were observed with coefficient of variation (CV) measures of evoked EPSCs, where baclofen application enhanced CV to the same degree in EAC3I MSNs and controls (NON EGFP baseline 0.21±0.05, 10 µM baclofen 0.56±0.07, 20 min washout 0.24±0.07; EGFP baseline 0.23±0.05, 10 µM baclofen 0.52±0.11, 20 min washout 0.31±0.06) [F (1, 9) = 0.0047; p = NS; Figure 3D]. Additionally, we found that baclofen significantly decreased the sEPSC frequency to a similar extent in both EAC3I MSNs and controls (NON EGFP baseline 100±22.4%, 10 µM baclofen 37.8±11.1%; EGFP baseline 100±33.2%, 10 µM baclofen 40.4±10.2%) [F (1, 9) = 0.0025; p = NS; Figure 3F].

**Figure 4:**
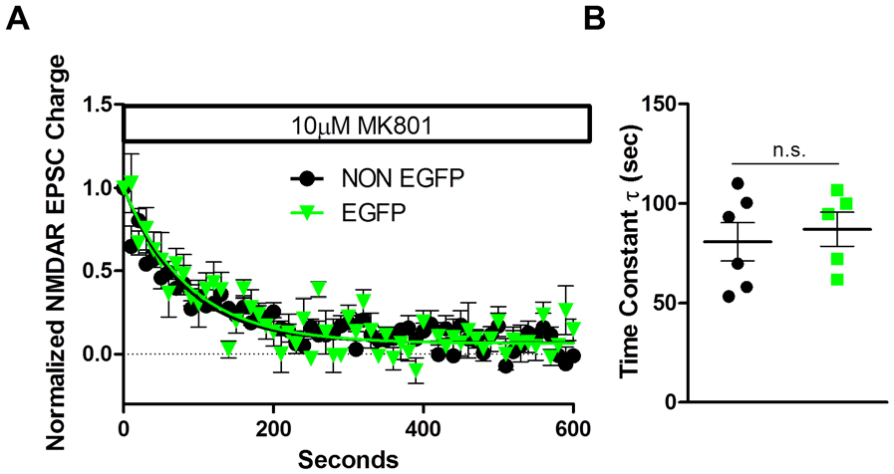
Dorsal lateral striatum MSN CaMKII inhibition does not alter the level of glutamate at the cleft. (A) Normalized average NMDAR-mediated EPSC Charge (AUC) measured with 0.1 Hz stimulation in the presence of 10 µM Mk-801 over time for EAC3I-containing and EAC3I-lacking MSNs. (B) The rate of NMDA-mediated EPSC decay is best fit with a single exponential decay function. The time constant (tau) in seconds was not significantly different between groups (NON EGFP: n = 6; EGFP: n = 5; p = 0.65). All neurons held at -70 mV to relieve NMDAR-dependent voltage blockade by magnesium.

Changes in s/mEPSC frequency could also theoretically be produced by changes in synaptic glutamate concentration. To address this possibility, we utilized MK-801, an uncompetitive, activity-dependent and irreversible antagonist of NMDARs [64]. After obtaining a stable baseline of evoked NMDAR-mediated EPSCs at +40 mV in the presence of picrotoxin and NBQX, the stimulator was switched off and 10 µM MK-801 was applied to the slice. After eight to ten minutes to allow the drug to equilibrate in the bath the stimulator was turned back on and the subsequent rate of inhibition of the NMDAR-mediated EPSC was calculated. The rate of inhibition was not significantly different between non-EGFP and EAC3I MSNs (tau in seconds, NON EGFP 80.82±9.65; EGFP 87.07±8.59) [t (9) = 0.6469; p = NS; Figure 4A, 4B], suggesting that the levels of glutamate at both synapses is not significantly different. Together, the lack of effect of CaMKII inhibition on PPR, baclofen modulation or MK-801 rate of blockade point to a postsynaptic mechanism for the decreased sEPSC frequency in the CaMKII-inhibited cells, which we interpret as a decrease in the number of functional synapses.

### CaMKII inhibition does not alter dendritic spine density, but reduces dendritic length and complexity

**Figure 5:**
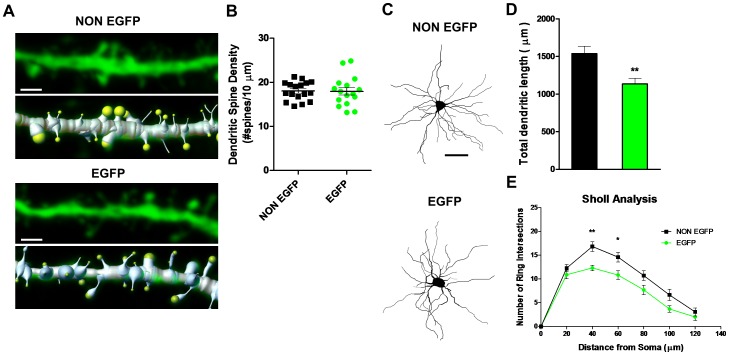
CaMKII inhibition does not alter dendritic spine density, but decreases dendritic length and complexity. Rendering and quantification of a confocal image of a Lucifer yellow filled dendritic segment (80–100 µm from the cell soma) from a MSN from the dorsal lateral striatum. (A) (top) Confocal image of EAC3I-lacking MSN (NON EGFP) and the Imaris dendrite and spine model overlaid from segment above. (below) Same as above, but for an EAC3I-containg MSN segment. Scale bars 1.5 µm. Fluorescent signal (green) pertains to Lucifer yellow fill. (B) Average dendritic spine density (number of spines/10 µm) scatter plot for each neuron. NON EGFP n = 17, EGFP n = 16; p = 0.93. (C) Neuronal reconstructions of representative EAC3I-lacking (NONEGFP) and EAC3I-containing (EGFP) dorsal striatal MSNs. Scale bar 50 µm. (D) Average total dendritic length in EAC3I-lacking (black) and EAC3I-containing (green) MSNs. (E) Sholl analysis of dendritic complexity in EAC3I-lacking (black) and EAC3I-containing MSNs (green). **p<0.01, *p<0.05; error bars represent SEM.

We next examined dendritic spine density, dendritic length and branching complexity in CaMKII-inhibited versus neighboring non-inhibited cells. Previous work has suggested a correlation between changes in mEPSC frequency and dendritic spine density [65], [66], [67], [68], although this is not always the case [69]. Additionally, CaMKII has been shown to modulate dendritic length in the hippocampus [70]. There was no difference in spine density related to expression of the transgene (NON EGFP 18.03±0.52 spines per 10 µm vs.; EGFP 17.95±0.85) [t (31) = 0.087; p = NS; Figure 5A, 5B]. However, we observed a significant reduction in total dendritic length (NON EGFP 1538±99 µm vs.; EGFP 1134±78) [t (31) = 3.158; p = 0.0035; Figure 5C, 5D]. Sholl analysis revealed a significant overall decrease in dendritic branching (F (1,31) = 28.55; p<0.0001), with Bonferroni post-hoc analyses revealing specific significant decreases at 40 and 60 µm distal to the cell soma (Figure 5E).

### GluA1 KO mimics EAC3I decrease in sEPSC frequency

**Figure 6:**
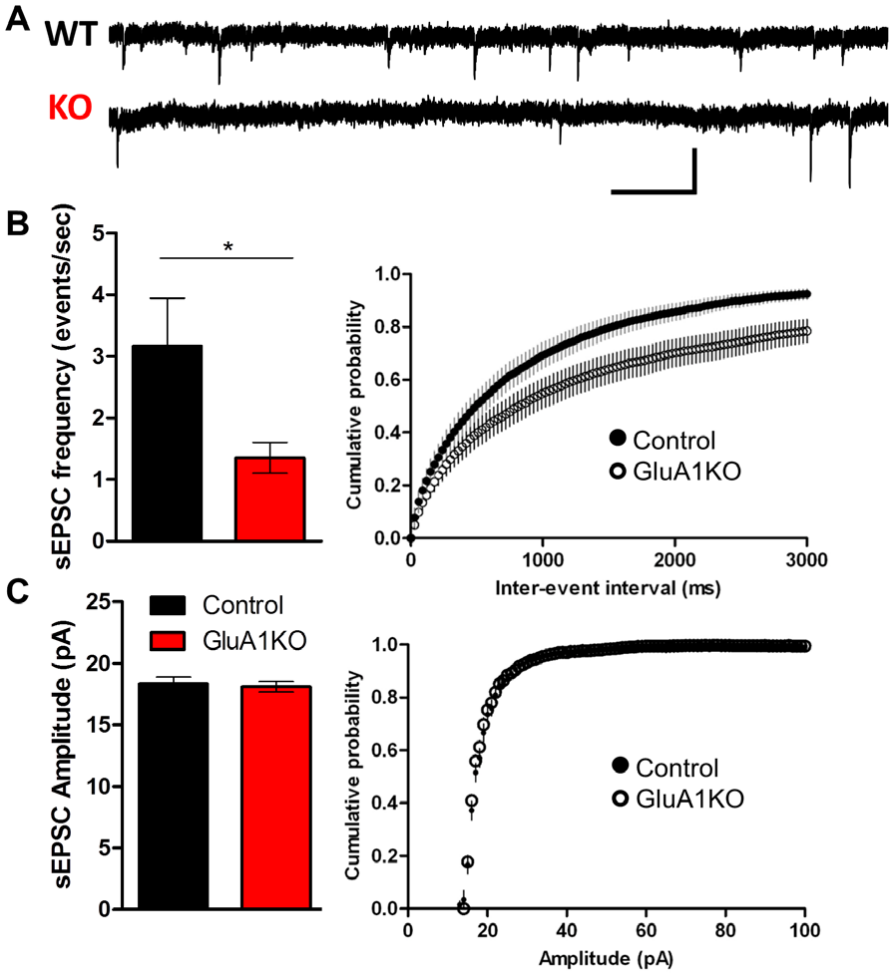
GluA1KO mice mimic the EAC3I mice decrease in sEPSC frequency. (A) Example traces of sEPSCs collected from dorsal lateral striatum MSNs in Wt (top) and GluA1KO (bottom). Scale bars 0.5 sec, 30 pA (Control: n = 15; GluA1KO: n = 18; p = 0.013). (B) (left) Average sEPSC frequency in GluA1KO versus controls. (right) Cumulative probability graph of inter-event interval. (C) (left) Average sEPSC amplitude in GluA1KO versus controls. (right) Cumulative probability graph of amplitude (Control: n = 15; GluA1KO: n = 18; p = 0.72). * P<0.05 ; error bars represent SEM. All MSNs were held at −70 mV.

The AMPAR GluA1 subunit is critical for activity-dependent postsynaptic strengthening of excitatory synapses, and is inserted into the synaptic membrane in a CaMKII-dependent process in hippocampal neurons [26]. Mice lacking the GluA1 subunit of the AMPAR have deficits in CA1 LTP [57] (but see [71]) and deficits in learning and memory [72], [73], [74], [75]. Additionally, reductions in CaMKIIα mRNA and protein levels are seen in the hippocampus of GluA1 KO animals [76]. If the CaMKII inhibition-dependent reduction in functional glutamatergic synapses on striatal MSNs is due to defects in synaptic GluA1 insertion, then we predicted that GluA1 knockout mice should mimic EAC3I mice in terms of sEPSC frequency and amplitude. Thus, we measured sEPSC frequency and amplitude in adult GluA1 KO versus control mice in the dorsal lateral striatum. We found that the loss of the GluA1 receptor also led to a significant reduction in sEPSC frequency (in Hz, control 3.2±0.8; GluA1KO 1.4±0.3) [U (31) = 66; p = 0.0133; Figure 6A, 6B], but not sEPSC amplitude (in pA, control 18.3±0.5; GluA1KO 18.1±0.4) [t (31) = 0.3576; p = N.S.; Figure 6A, 6C]. The similarity in synaptic outcomes of the GluA1 KO and EAC3I expression reinforces the idea that a common pathway has been affected.

### MSN CaMKII inhibition leads to enhanced intrinsic excitability

**Figure 7:**
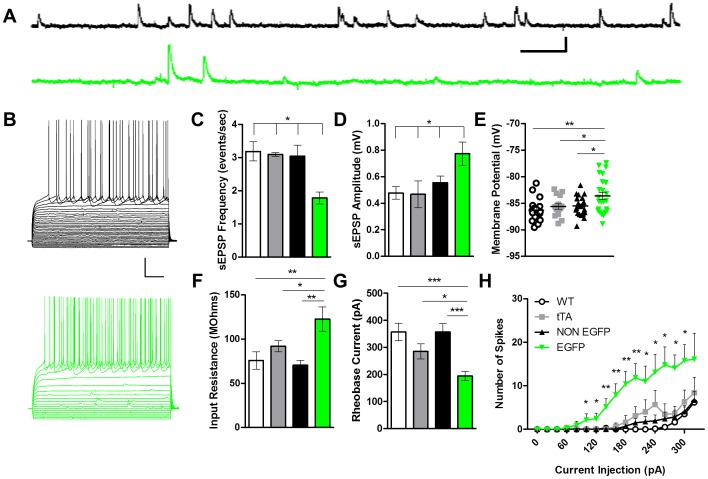
CaMKII inhibition enhances MSN intrinsic excitability. (A) Traces of EAC3I-lacking (NON EGFP, black) and EAC3I-containing MSNs (EGFP, green) sEPSPs recorded at −85 mV. Scale bars 0.6 mV, 200 ms. (B) Traces of EAC3I-lacking (black) and EAC3I-containing MSNs (green) with 20 pA hyperpolarizing and depolarizing current injections (−120 pA to +100 pA above AP threshold, 20 pA steps). Scale bars 200 ms, 20 mV. (C) Average sEPSP frequency in EAC3I-containing MSNs versus controls (Wt: n = 4; tTA: n = 4; NON EGFP: n = 9; versus EGFP: n = 6; p = 0.015). (D) Average sEPSP amplitude (current clamped at −85 mV) in EAC3I-containing MSNs versus controls (Wt: n = 4; tTA: n = 4; NON EGFP: n = 9; versus EGFP: n = 6; p = 0.037). (E) Resting membrane potential (RMP) (mV) of EAC3I-containing and control MSNs (Wt: n = 18; tTA: n = 15; NON EGFP: n = 22; versus EGFP: n = 24; p = 0.0043). (F) Input resistance of EAC3I-containing and control MSNs (Wt: n = 18; tTA: n = 15; NON EGFP: n = 22; versus EGFP: n = 24; p = 0.0002). (G) Rheobase current injection or current injection to reach 1^st^ AP in EAC3I-containing and control MSNs (Wt: n = 18; tTA: n = 15; NON EGFP: n = 22; versus EGFP: n = 24; p<0.0001). (H) Firing frequency (Hz) after 4 sweeps (20 pA steps) following threshold firing in EAC3I-containing and control MSNs (Wt: n = 18; tTA: n = 15; NON EGFP: n = 22; versus EGFP: n = 24; p = 0.0023). * P<0.05; ** P<0.01; *** *P<0.001;* error bars represent SEM.

To further examine the impact of CaMKII inhibition on physiological responses of dorsal striatal MSNs, we next examined excitatory drive and excitability of these cells under current clamp conditions. As expected from our voltage clamp experiments with sEPSCs, we also observed a robust decrease in the frequency of sEPSPs in the CaMKII inhibited cells versus control neurons in current clamp (in Hz, Wt 3.18±0.29; tTA 3.09±0.06; NON EGFP 3.04±0.33; EGFP 1.78±0.18) [F (3, 19) = 4.704; p = 0.0154; Figure 7A, 7C]. Surprisingly, however, we also observed a significant increase in sEPSP amplitude in the CaMKII-inhibited cells versus controls (in mV, Wt 0.48±0.05; tTA 0.47±0.10; NON EGFP 0.55±0.05; EGFP 0.77±0.09) [F (3, 19) = 3.605; p = 0.0367; Figure 7A, 7D]. As similar effects were not observed with sEPSC amplitudes, this suggested a change in excitability of EAC3I neurons. In order to further test this idea we measured basal intrinsic excitability. EGFP cells possessed a significantly more depolarized resting membrane potential (in mV, Wt −86.30±0.50; tTA −85.61±0.50; NON EGFP −85.52±0.38; EGFP −83.61±0.68) [H (3, 78) = 8.588; p = 0.0353; Figure 7E] and had significantly increased input resistance compared to control cells (in MΩ, Wt 75.85±9.90; tTA 91.98±6.22; NON EGFP 70.48±5.54; EGFP 122.65±13.81) [H (3, 79) = 19.89; p = 0.0002; Figure 7F]. This suggests that CaMKII inhibition moves the resting membrane potential closer to firing threshold and increases membrane resistance to enhance the propagation of depolarizing current from distal MSN dendrites. Next we examined the voltage responses to differing current injections. While injecting minimal current to maintain resting membrane potential at −85 mV, a series of hyperpolarizing and depolarizing current injections were given in 20 pA steps. The threshold for 1^st^ AP or rheobase current injection was significantly lower in the CaMKII inhibited MSNs versus control (in pA, Wt 357±32; tTA 285±28; NON EGFP 357±30; EGFP 194±17) [F (3, 78) = 9.393; p<0.0001; Figure 7G]. The firing threshold was not significantly different amongst groups (in mV, Wt −36.4±1.3; tTA −35.3±1.5; NON EGFP −33.4±0.8; EGFP −37.0±0.9) [F (3, 78) = 2.516; p = 0.0649], suggesting that input resistance changes are a major contributor to changes in rheobase. Also CaMKII inhibited cells exhibited increased spiking over a range of suprathreshold current injections [F (3,60) = 5.425; p = 0.0023; Figure 7H] versus control, reflecting a decrease in the interspike interval. Taken together, these data show that CaMKII inhibition enhances the intrinsic excitability of MSNs.

## Discussion

We present converging lines of evidence that dorsal striatal MSN CaMKII inhibition decreases functional synapse number and increases intrinsic excitability. Inhibition of CaMKII in MSNs leads to a decrease in sEPSC frequency, without a change in release probability, glutamate levels at the synaptic cleft or dendritic spine density. These observations are consistent with a decrease in the number of functional synapses. In addition to changes in excitatory transmission, inhibition of CaMKII leads to an enhancement of MSN intrinsic excitability. These data suggest that CaMKII coordinates opposing regulation of excitatory transmission and intrinsic excitability in MSNs, serving as a cellular rheostat.

CaMKII inhibitors such as KN62 and KN93 have been useful tools in probing CaMKII functions, but these drugs also inhibit voltage-gated K^+^ and Ca^2+^ channels [77], [78], and do not inhibit the autonomous activity of Thr286-autophosphorylated CaMKII [79], [80]. In addition, in dendritic spines where the concentration of calmodulin and CaMKII are extremely high (∼100 µM) [81], [82], KN-62 (10 µm) only partially decreases CaMKII activity [18]. The EAC3I peptide we used inhibits all isoforms of CaMKII, including CaM-stimulated and autonomous activity, with low micromolar potency [52], [56], [83]. EAC3-I is ≥100-fold selective for CaMKII over protein kinase C, CaM Kinase I or CaM kinase IV [52], [54]. The AC3-I peptide sequence differs from another highly selective CaMKII-inhibitor, AIP, by only one amino acid residue [55]. It is also important to consider the localization of CaMKII inhibition when interpreting these results. CaMKII is highly expressed in dopamine terminals, which densely innervate the striatum, where it stimulates dopamine efflux via the dopamine transporter in the presence of amphetamine [51]. In addition, CaMKII is present in glutamatergic projections, which form the presynaptic terminal onto MSN spines and dendrites [33], [51], where it may modulate release events [39], [43], [44], [45]. Our transgenic strategy resulted in the selective expression of the CaMKII inhibitor in the postsynaptic MSN, where it cannot directly affect the function of the glutamatergic and dopaminergic terminals, consistent with the lack of change in glutamate release parameters in EAC3I MSNs (Figures 3, 4).

We demonstrated that CaMKII inhibition decreases s/mEPSC frequency with no changes in presynaptic function. The lack of a bimodal distribution in the s/mEPSC frequency data suggests that CaMKII inhibition similarly affects both direct and indirect pathway MSNs. Changes in s/mEPSC frequency are traditionally interpreted as alterations in presynaptic quantal content; the product of changes in release probability or synapse number. However, multiple lines of evidence suggest that presynaptic function is unaltered. Together these data suggest that CaMKII inhibition decreases functional synapse number. One possibility is that CaMKII inhibition produces a loss of functional synaptic connections. Alternatively, there could be an increase in the number of silent synapses which contain NMDARs but no AMPARs and are typically abundant early in development [84], [85], [86], [87], [88], [89]. A further possibility is that CaMKII inhibition could increase the numbers of silent modules of synapses or increase the number of AMPAR-lacking subregions of the synapse due to local nature of basal synaptic transmission [90], [91]. In the hippocampus, silent synapses can be unsilenced following NMDAR activation by the introduction of new AMPAR to the synapse, underlying a common mechanism of LTP of synaptic glutamatergic transmission [26], [27], [92], [93]. Previous research has suggested a direct role for CaMKII in the unsilencing of synapses in the hippocampus [94], [95], [96], [97]. Additionally, CaMKII is required for the formation of new synapses and/or morphological growth following hippocampal LTP induction [18], [98], [99], [100].

Striatal CaMKII inhibition did not alter dendritic spine density, suggesting that the decrease in s/mEPSC frequency could best be explained by some spines lacking active presynaptic terminals or increased numbers of silent synapses. However, the significant increase in the input resistance of EAC3I-positive MSNs should enhance sampling of mEPSCs from more distal dendritic sites, potentially increasing s/mEPSC frequency. These data together suggest that decreases in s/mEPSC frequency in EAC3-I MSNs are potentially underestimated. Alternatively, some of these effects may be due to decreased dendritic length and complexity seen in EAC3I-positive MSNs. However, it is not clear whether distal MSN synapses are sampled in our s/mEPSC analyses due to cable filtering effects previously reported [101]. Hippocampal CaMKIIβ has been shown to modulate dendritic length and branching as well as synapse number [70]. These results suggest that CaMKII plays important roles in modeling MSN dendritic morphology. The CaMKII alpha promoter fragment that drives EAC3I expression reportedly turns on around P5 [102], [103], [104], [105]. This would lead to inhibition of CaMKII in early postnatal development and continuing into adulthood. The effects of this longer term genetic CaMKII inhibition contrasts with the typically minimal effects of acute, short term application of a related CaMKII inhibitor peptide, AIP, on basal glutamatergic transmission in the CA1 and CA3 region of the hippocampus [106], [107], [108], [109]. Conversely, expression of CaMKII inhibitor peptides CaMKIIN or AIP over 2–6 days reduced hippocampal CA1 AMPAR-mediated, but not NMDAR-mediated EPSCs [110]. Another report suggests a CaMKII inhibitor peptide, CN19, persistently decreased hippocampal CA1 field EPSPs or EPSCs amplitudes at higher concentrations disrupting the CaMKII/NMDAR complex [111]. These data suggest that disruption of the CaMKII/NMDAR complex, a complex that increases following strong synaptic stimulation [112] and is necessary for LTP [113], [114], may offer an alternative mechanism underlying our observed effects.

Endogenous CaMKIIα levels peak around 3 weeks postnatally, which is a crucial time for synaptogenesis and synapse maturation [104], [105], [115]. The apparent decrease in functional synaptic connections in CaMKII inhibited MSNs in adulthood may have four possible explanations: 1) CaMKII activity is necessary for the normal unsilencing of synapses in the adult; 2) Ongoing CaMKII activity is required to maintain functional synapses; or 3) CaMKII is needed early in synaptogenesis to turn initially silent synapses into functional ones; or 4) the CaMKII-mediated decrease in dendritic length may underlie the reduction in total synapse number. The decrease in sEPSC frequency in EAC3I MSNs was detected as early as three weeks postnatally, suggesting a large proportion of synapses were never unsilenced or perhaps never formed. In the hippocampus both electrophysiological and anatomical studies at light and electron microscopic levels suggest that in the first few weeks of life many synapses start as NMDAR-only synapses or silent synapses [116], [117], [118], [119], [120]. Synapse unsilencing involving the trafficking of new AMPARs (GluA4-containing) to the synapse in early postnatal development is dependent on activity, but is independent of CaMKII [121], [122]. Instead, PKA plays an important role early in postnatal development (<P9) in plasticity in the hippocampus being necessary and sufficient for GluA4 incorporation, but requiring additional CaMKII activity for GluA1 receptor incorporation [122], [123], [124]. These data suggest that the similar synaptic phenotypes of GluA1KO and EAC3I mice arise from disruption of a common mechanism. However, it is interesting that the phenotype of the EAC3I cells is also virtually identical to that recently reported for MSNs in SAPAP3 knockout mice [7], [125]. Intriguingly, SAPAP3 may be phosphorylated by CaMKII, possibly assisting in synaptic targeting of GluA1R [126]. Thus, it will be important for future studies to directly identify downstream MSN proteins regulated by CaMKII.

It is also possible that differences in the relative innervation of MSNs by cortical and thalamic inputs impacts the synaptic phenotypes. Indeed, differences in release probability have been observed between the two inputs [127], [128], [129]. Our measures of sEPSC and mEPSC frequency are likely comprised of both cortical and thalamic-mediated glutamate release, yet our data do not rule out the possibility that inhibition of MSN CaMKII may have a greater influence on one of these excitatory synapses over the other.

CaMKII inhibition also leads to alterations in intrinsic excitability that may serve to broadly counteract the reduced s/mEPSC frequency. Although we expected a decrease in sEPSP frequency in current clamp based on the sEPSC results, we observed significantly larger sEPSP amplitudes. This is likely due to enhanced intrinsic excitability. In CaMKII-inhibited neurons we observed more depolarized resting membrane potentials, increased membrane resistance, decreased rheobase current injection, and increased firing frequency. Autonomously active CaMKII has been shown to suppress neuronal excitability by increasing cell-surface expression of an A-type K^+^ channel, Kv4.2, via phosphorylation [30], [130], [131]. In addition, CaMKII inhibition in medial vestibular nucleus neurons increased intrinsic excitability via a reduction in BK-type calcium activated potassium currents [132]. Changes in Kv4.2 or BK activity following CaMKII inhibition could account for the differences in firing that we observed in EAC3I cells. However, modulation of Kir 2 channels may be responsible for the changes in input resistance and resting membrane potential [133]. A recent study showed that acute CaMKII inhibition in cortical cultures leads to increased excitability, but also increased cell death [134]. We did not note increased cell death, nor did membrane properties hint at unhealthy EAC3I-expressing cells. The differences in these studies may be attributed to the fact that MSNs are GABAergic cells bypassing potential excitotoxicity vulnerabilities seen with recurrent excitatory connections in the cortex. Alternatively, cortical cultures – which are often more excitable - may have a more difficult time regulating extracellular glutamate levels, something that is not as problematic in *ex vivo* slice preparations.

The opposing regulation of excitatory transmission and excitability observed in these studies suggests that CaMKII may serve as a molecular fulcrum to counterbalance changes in enhanced excitatory input with decreases in excitatory output. It is important to note in the present dataset we cannot rule out that possibility that one of these adaptations is compensatory to the other, rather than both being directly initiated by CaMKII inhibition. Regardless, this likely has important implications for the modulation of basal ganglia circuitry underlying habit learning, addiction and neurodegenerative disease. CaMKII plays a role in setting the number of functional synapses and therefore may provide a substrate for experience dependent plasticity in the striatum. Dorsal striatal CaMKII may be crucial early in postnatal development as well as in adulthood entraining new motor repertoires and refining synaptic connections as those motor skills are refined into habits later in life. With the inhibition of CaMKII leading to a decrease in the number of functional contacts, CaMKII may function in the dendritic and synaptic maturation processes, from nascent filopodia to mature dendritic spine [18], [99], or alternatively be important in maintaining existing synaptic connections. Further investigation will be needed to determine the precise role of CaMKII in striatal synaptic maturation.

## Supporting Information

Figure S1 **Striatal interneuron markers do not colocalize with EAC3I peptide.** (A) 20× confocal image of dorsal lateral striatum from an EAC3I mouse of endogenous EGFP expression (green) and striatal ChAT immunopositive interneurons (red, 0/70 ChAT positive neurons contained EGFP). Scale bar 100 µm, inset 50 µm. (B) Like A, but striatal parvalbumin immunopositive interneurons labeled (red, 0/37 parvalbumin positive neurons contained EGFP). Scale bar 100 µm, inset 50 µm. (C) Like A, but striatal NPY immunopositive interneurons labeled (red, 0/22 NPY positive neurons contained EGFP). Scale bar 100 µm, inset 50 µm. (D) Like A, but striatal calretinin immunopositive interneurons labeled (red, 0/15 calretinin positive neurons contained EGFP). Scale bar 100 µm, inset 50 µm.
(TIF)

## References

[1] Yin HH, Knowlton BJ (2006) The role of the basal ganglia in habit formation. Nat Rev Neurosci 7: 464–476. 16715055 10.1038/nrn1919

[2] Jenner P (2008) Molecular mechanisms of L-DOPA-induced dyskinesia. Nat Rev Neurosci 9: 665–677. 18714325 10.1038/nrn2471

[3] Kreitzer AC, Malenka RC (2008) Striatal plasticity and basal ganglia circuit function. Neuron 60: 543–554. 19038213 10.1016/j.neuron.2008.11.005 PMC2724179

[4] Milnerwood AJ, Raymond LA (2010) Early synaptic pathophysiology in neurodegeneration: insights from Huntington's disease. Trends Neurosci 33: 513–523. 20850189 10.1016/j.tins.2010.08.002

[5] Redgrave P, Rodriguez M, Smith Y, Rodriguez-Oroz MC, Lehericy S, et al (2010) Goal-directed and habitual control in the basal ganglia: implications for Parkinson's disease. Nat Rev Neurosci 11: 760–772. 20944662 10.1038/nrn2915 PMC3124757

[6] Luscher C, Malenka RC (2011) Drug-evoked synaptic plasticity in addiction: from molecular changes to circuit remodeling. Neuron 69: 650–663. 21338877 10.1016/j.neuron.2011.01.017 PMC4046255

[7] Wan Y, Feng G, Calakos N (2011) Sapap3 deletion causes mGluR5-dependent silencing of AMPAR synapses. J Neurosci 31: 16685–16691. 22090495 10.1523/JNEUROSCI.2533-11.2011 PMC3475185

[8] Yang XW, Lu XH (2011) Molecular and cellular basis of obsessive-compulsive disorder-like behaviors: emerging view from mouse models. Curr Opin Neurol 24: 114–118. 21386675 10.1097/WCO.0b013e32834451fb

[9] Erondu NE, Kennedy MB (1985) Regional distribution of type II Ca2+/calmodulin-dependent protein kinase in rat brain. J Neurosci 5: 3270–3277. 4078628 10.1523/JNEUROSCI.05-12-03270.1985 PMC6565219

[10] Lisman J, Schulman H, Cline H (2002) The molecular basis of CaMKII function in synaptic and behavioural memory. Nat Rev Neurosci 3: 175–190. 11994750 10.1038/nrn753

[11] Fukunaga K, Goto S, Miyamoto E (1988) Immunohistochemical localization of Ca2+/calmodulin-dependent protein kinase II in rat brain and various tissues. J Neurochem 51: 1070–1078. 3047316 10.1111/j.1471-4159.1988.tb03070.x

[12] Cheng D, Hoogenraad CC, Rush J, Ramm E, Schlager MA, et al (2006) Relative and absolute quantification of postsynaptic density proteome isolated from rat forebrain and cerebellum. Mol Cell Proteomics 5: 1158–1170. 16507876 10.1074/mcp.D500009-MCP200

[13] Baucum AJ 2nd, Strack S, Colbran RJ (2012) Age-dependent targeting of protein phosphatase 1 to Ca2+/calmodulin-dependent protein kinase II by spinophilin in mouse striatum. PLoS One 7: e31554. 22348105 10.1371/journal.pone.0031554 PMC3278457

[14] Silva AJ, Stevens CF, Tonegawa S, Wang Y (1992) Deficient hippocampal long-term potentiation in alpha-calcium-calmodulin kinase II mutant mice. Science 257: 201–206. 1378648 10.1126/science.1378648

[15] Hinds HL, Tonegawa S, Malinow R (1998) CA1 long-term potentiation is diminished but present in hippocampal slices from alpha-CaMKII mutant mice. Learn Mem 5: 344–354. 10454359 PMC311262

[16] Giese KP, Fedorov NB, Filipkowski RK, Silva AJ (1998) Autophosphorylation at Thr286 of the alpha calcium-calmodulin kinase II in LTP and learning. Science 279: 870–873. 9452388 10.1126/science.279.5352.870

[17] Lucchesi W, Mizuno K, Giese KP (2011) Novel insights into CaMKII function and regulation during memory formation. Brain Res Bull 85: 2–8. 21070840 10.1016/j.brainresbull.2010.10.009

[18] Lee SJ, Escobedo-Lozoya Y, Szatmari EM, Yasuda R (2009) Activation of CaMKII in single dendritic spines during long-term potentiation. Nature 458: 299–304. 19295602 10.1038/nature07842 PMC2719773

[19] Wayman GA, Lee YS, Tokumitsu H, Silva AJ, Soderling TR (2008) Calmodulin-kinases: modulators of neuronal development and plasticity. Neuron 59: 914–931. 18817731 10.1016/j.neuron.2008.08.021 PMC2664743

[20] Barria A, Derkach V, Soderling T (1997) Identification of the Ca2+/calmodulin-dependent protein kinase II regulatory phosphorylation site in the alpha-amino-3-hydroxyl-5-methyl-4-isoxazole-propionate-type glutamate receptor. J Biol Chem 272: 32727–32730. 9407043 10.1074/jbc.272.52.32727

[21] Mammen AL, Kameyama K, Roche KW, Huganir RL (1997) Phosphorylation of the alpha-amino-3-hydroxy-5-methylisoxazole4-propionic acid receptor GluR1 subunit by calcium/calmodulin-dependent kinase II. J Biol Chem 272: 32528–32533. 9405465 10.1074/jbc.272.51.32528

[22] Derkach V, Barria A, Soderling TR (1999) Ca2+/calmodulin-kinase II enhances channel conductance of alpha-amino-3-hydroxy-5-methyl-4-isoxazolepropionate type glutamate receptors. Proc Natl Acad Sci U S A 96: 3269–3274. 10077673 10.1073/pnas.96.6.3269 PMC15931

[23] Lee HK, Barbarosie M, Kameyama K, Bear MF, Huganir RL (2000) Regulation of distinct AMPA receptor phosphorylation sites during bidirectional synaptic plasticity. Nature 405: 955–959. 10879537 10.1038/35016089

[24] Benke TA, Luthi A, Isaac JT, Collingridge GL (1998) Modulation of AMPA receptor unitary conductance by synaptic activity. Nature 393: 793–797. 9655394 10.1038/31709

[25] Strack S, McNeill RB, Colbran RJ (2000) Mechanism and regulation of calcium/calmodulin-dependent protein kinase II targeting to the NR2B subunit of the N-methyl-D-aspartate receptor. J Biol Chem 275: 23798–23806. 10764765 10.1074/jbc.M001471200

[26] Hayashi Y, Shi SH, Esteban JA, Piccini A, Poncer JC, et al (2000) Driving AMPA receptors into synapses by LTP and CaMKII: requirement for GluR1 and PDZ domain interaction. Science 287: 2262–2267. 10731148 10.1126/science.287.5461.2262

[27] Shi SH, Hayashi Y, Petralia RS, Zaman SH, Wenthold RJ, et al (1999) Rapid spine delivery and redistribution of AMPA receptors after synaptic NMDA receptor activation. Science 284: 1811–1816. 10364548 10.1126/science.284.5421.1811

[28] Lledo PM, Zhang X, Sudhof TC, Malenka RC, Nicoll RA (1998) Postsynaptic membrane fusion and long-term potentiation. Science 279: 399–403. 9430593 10.1126/science.279.5349.399

[29] Shi S, Hayashi Y, Esteban JA, Malinow R (2001) Subunit-specific rules governing AMPA receptor trafficking to synapses in hippocampal pyramidal neurons. Cell 105: 331–343. 11348590 10.1016/s0092-8674(01)00321-x

[30] Varga AW, Yuan LL, Anderson AE, Schrader LA, Wu GY, et al (2004) Calcium-calmodulin-dependent kinase II modulates Kv4.2 channel expression and upregulates neuronal A-type potassium currents. J Neurosci 24: 3643–3654. 15071113 10.1523/JNEUROSCI.0154-04.2004 PMC6729731

[31] Kourrich S, Klug JR, Mayford M, Thomas MJ (2012) AMPAR-independent effect of striatal alphaCaMKII promotes the sensitization of cocaine reward. J Neurosci 32: 6578–6586. 22573680 10.1523/JNEUROSCI.6391-11.2012 PMC3448780

[32] Sik A, Hajos N, Gulacsi A, Mody I, Freund TF (1998) The absence of a major Ca2+ signaling pathway in GABAergic neurons of the hippocampus. Proc Natl Acad Sci U S A 95: 3245–3250. 9501248 10.1073/pnas.95.6.3245 PMC19727

[33] Liu XB, Jones EG (1996) Localization of alpha type II calcium calmodulin-dependent protein kinase at glutamatergic but not gamma-aminobutyric acid (GABAergic) synapses in thalamus and cerebral cortex. Proc Natl Acad Sci U S A 93: 7332–7336. 8692993 10.1073/pnas.93.14.7332 PMC38984

[34] Jones EG, Huntley GW, Benson DL (1994) Alpha calcium/calmodulin-dependent protein kinase II selectively expressed in a subpopulation of excitatory neurons in monkey sensory-motor cortex: comparison with GAD-67 expression. J Neurosci 14: 611–629. 8301355 10.1523/JNEUROSCI.14-02-00611.1994 PMC6576801

[35] Picconi B, Gardoni F, Centonze D, Mauceri D, Cenci MA, et al (2004) Abnormal Ca2+-calmodulin-dependent protein kinase II function mediates synaptic and motor deficits in experimental parkinsonism. J Neurosci 24: 5283–5291. 15190099 10.1523/JNEUROSCI.1224-04.2004 PMC6729313

[36] Wiltgen BJ, Law M, Ostlund S, Mayford M, Balleine BW (2007) The influence of Pavlovian cues on instrumental performance is mediated by CaMKII activity in the striatum. Eur J Neurosci 25: 2491–2497. 17445244 10.1111/j.1460-9568.2007.05487.x

[37] Stein IS, Hell JW (2010) CaMKII hunkers down on the muscarinic M4 receptor to help curb cocaine-induced hyperlocomotion. EMBO J 29: 1943–1945. 20551968 10.1038/emboj.2010.105 PMC2892364

[38] Stefani G, Onofri F, Valtorta F, Vaccaro P, Greengard P, et al (1997) Kinetic analysis of the phosphorylation-dependent interactions of synapsin I with rat brain synaptic vesicles. J Physiol 504 Pt 3: 501–515. 9401959 10.1111/j.1469-7793.1997.501bd.x PMC1159955

[39] Chi P, Greengard P, Ryan TA (2001) Synapsin dispersion and reclustering during synaptic activity. Nat Neurosci 4: 1187–1193. 11685225 10.1038/nn756

[40] Waxham MN, Malenka RC, Kelly PT, Mauk MD (1993) Calcium/calmodulin-dependent protein kinase II regulates hippocampal synaptic transmission. Brain Res 609: 1–8. 8389645 10.1016/0006-8993(93)90847-g

[41] Lin JW, Sugimori M, Llinas RR, McGuinness TL, Greengard P (1990) Effects of synapsin I and calcium/calmodulin-dependent protein kinase II on spontaneous neurotransmitter release in the squid giant synapse. Proc Natl Acad Sci U S A 87: 8257–8261. 1978321 10.1073/pnas.87.21.8257 PMC54934

[42] Llinas R, McGuinness TL, Leonard CS, Sugimori M, Greengard P (1985) Intraterminal injection of synapsin I or calcium/calmodulin-dependent protein kinase II alters neurotransmitter release at the squid giant synapse. Proc Natl Acad Sci U S A 82: 3035–3039. 2859595 10.1073/pnas.82.9.3035 PMC397701

[43] Hojjati MR, van Woerden GM, Tyler WJ, Giese KP, Silva AJ, et al (2007) Kinase activity is not required for alphaCaMKII-dependent presynaptic plasticity at CA3-CA1 synapses. Nat Neurosci 10: 1125–1127. 17660813 10.1038/nn1946 PMC2804046

[44] Jiang X, Lautermilch NJ, Watari H, Westenbroek RE, Scheuer T, et al (2008) Modulation of CaV2.1 channels by Ca2+/calmodulin-dependent protein kinase II bound to the C-terminal domain. Proc Natl Acad Sci U S A 105: 341–346. 18162541 10.1073/pnas.0710213105 PMC2224214

[45] Elgersma Y, Fedorov NB, Ikonen S, Choi ES, Elgersma M, et al (2002) Inhibitory autophosphorylation of CaMKII controls PSD association, plasticity, and learning. Neuron 36: 493–505. 12408851 10.1016/s0896-6273(02)01007-3

[46] Carlier E, Dargent B, De Waard M, Couraud F (2000) Na(+) channel regulation by calmodulin kinase II in rat cerebellar granule cells. Biochem Biophys Res Commun 274: 394–399. 10913349 10.1006/bbrc.2000.3145

[47] Wagner S, Dybkova N, Rasenack EC, Jacobshagen C, Fabritz L, et al (2006) Ca2+/calmodulin-dependent protein kinase II regulates cardiac Na+ channels. J Clin Invest 116: 3127–3138. 17124532 10.1172/JCI26620 PMC1654201

[48] Yamauchi T, Nakata H, Fujisawa H (1981) A new activator protein that activates tryptophan 5-monooxygenase and tyrosine 3-monooxygenase in the presence of Ca2+-, calmodulin-dependent protein kinase. Purification and characterization. J Biol Chem 256: 5404–5409. 6113235

[49] Atkinson J, Richtand N, Schworer C, Kuczenski R, Soderling T (1987) Phosphorylation of purified rat striatal tyrosine hydroxylase by Ca2+/calmodulin-dependent protein kinase II: effect of an activator protein. J Neurochem 49: 1241–1249. 2887635 10.1111/j.1471-4159.1987.tb10016.x

[50] Binda F, Dipace C, Bowton E, Robertson SD, Lute BJ, et al (2008) Syntaxin 1A interaction with the dopamine transporter promotes amphetamine-induced dopamine efflux. Mol Pharmacol 74: 1101–1108. 18617632 10.1124/mol.108.048447 PMC2728020

[51] Fog JU, Khoshbouei H, Holy M, Owens WA, Vaegter CB, et al (2006) Calmodulin kinase II interacts with the dopamine transporter C terminus to regulate amphetamine-induced reverse transport. Neuron 51: 417–429. 16908408 10.1016/j.neuron.2006.06.028

[52] Braun AP, Schulman H (1995) A non-selective cation current activated via the multifunctional Ca(2+)-calmodulin-dependent protein kinase in human epithelial cells. J Physiol 488 Pt 1: 37–55. 8568664 10.1113/jphysiol.1995.sp020944 PMC1156699

[53] Wu Y, Temple J, Zhang R, Dzhura I, Zhang W, et al (2002) Calmodulin kinase II and arrhythmias in a mouse model of cardiac hypertrophy. Circulation 106: 1288–1293. 12208807 10.1161/01.cir.0000027583.73268.e7

[54] Patel R, Holt M, Philipova R, Moss S, Schulman H, et al (1999) Calcium/calmodulin-dependent phosphorylation and activation of human Cdc25-C at the G2/M phase transition in HeLa cells. J Biol Chem 274: 7958–7968. 10075693 10.1074/jbc.274.12.7958

[55] Vest RS, Davies KD, O'Leary H, Port JD, Bayer KU (2007) Dual mechanism of a natural CaMKII inhibitor. Mol Biol Cell 18: 5024–5033. 17942605 10.1091/mbc.E07-02-0185 PMC2096578

[56] Zhang R, Khoo MS, Wu Y, Yang Y, Grueter CE, et al (2005) Calmodulin kinase II inhibition protects against structural heart disease. Nat Med 11: 409–417. 15793582 10.1038/nm1215

[57] Zamanillo D, Sprengel R, Hvalby O, Jensen V, Burnashev N, et al (1999) Importance of AMPA receptors for hippocampal synaptic plasticity but not for spatial learning. Science 284: 1805–1811. 10364547 10.1126/science.284.5421.1805

[58] Kawaguchi Y, Wilson CJ, Emson PC (1989) Intracellular recording of identified neostriatal patch and matrix spiny cells in a slice preparation preserving cortical inputs. J Neurophysiol 62: 1052–1068. 2585039 10.1152/jn.1989.62.5.1052

[59] Mayford M, Bach ME, Huang YY, Wang L, Hawkins RD, et al (1996) Control of memory formation through regulated expression of a CaMKII transgene. Science 274: 1678–1683. 8939850 10.1126/science.274.5293.1678

[60] Kaufman WL, Kocman I, Agrawal V, Rahn HP, Besser D, et al (2008) Homogeneity and persistence of transgene expression by omitting antibiotic selection in cell line isolation. Nucleic Acids Res 36: e111. 18682524 10.1093/nar/gkn508 PMC2553579

[61] Bejar R, Yasuda R, Krugers H, Hood K, Mayford M (2002) Transgenic calmodulin-dependent protein kinase II activation: dose-dependent effects on synaptic plasticity, learning, and memory. J Neurosci 22: 5719–5726. 12097524 10.1523/JNEUROSCI.22-13-05719.2002 PMC6758231

[62] Zucker RS, Regehr WG (2002) Short-term synaptic plasticity. Annu Rev Physiol 64: 355–405. 11826273 10.1146/annurev.physiol.64.092501.114547

[63] Lei S, McBain CJ (2003) GABA B receptor modulation of excitatory and inhibitory synaptic transmission onto rat CA3 hippocampal interneurons. J Physiol 546: 439–453. 12527730 10.1113/jphysiol.2002.034017 PMC2342507

[64] Huettner JE, Bean BP (1988) Block of N-methyl-D-aspartate-activated current by the anticonvulsant MK-801: selective binding to open channels. Proc Natl Acad Sci U S A 85: 1307–1311. 2448800 10.1073/pnas.85.4.1307 PMC279756

[65] Day M, Wang Z, Ding J, An X, Ingham CA, et al (2006) Selective elimination of glutamatergic synapses on striatopallidal neurons in Parkinson disease models. Nat Neurosci 9: 251–259. 16415865 10.1038/nn1632

[66] Verpelli C, Dvoretskova E, Vicidomini C, Rossi F, Chiappalone M, et al (2011) Importance of Shank3 protein in regulating metabotropic glutamate receptor 5 (mGluR5) expression and signaling at synapses. J Biol Chem 286: 34839–34850. 21795692 10.1074/jbc.M111.258384 PMC3186429

[67] Lu Y, Zha XM, Kim EY, Schachtele S, Dailey ME, et al (2011) A kinase anchor protein 150 (AKAP150)-associated protein kinase A limits dendritic spine density. J Biol Chem 286: 26496–26506. 21652711 10.1074/jbc.M111.254912 PMC3143614

[68] Fu WY, Chen Y, Sahin M, Zhao XS, Shi L, et al (2007) Cdk5 regulates EphA4-mediated dendritic spine retraction through an ephexin1-dependent mechanism. Nat Neurosci 10: 67–76. 17143272 10.1038/nn1811

[69] Ding JB, Oh WJ, Sabatini BL, Gu C (2012) Semaphorin 3E-Plexin-D1 signaling controls pathway-specific synapse formation in the striatum. Nat Neurosci 15: 215–223. 10.1038/nn.3003 PMC3267860 22179111

[70] Fink CC, Bayer KU, Myers JW, Ferrell JE Jr, Schulman H, et al (2003) Selective regulation of neurite extension and synapse formation by the beta but not the alpha isoform of CaMKII. Neuron 39: 283–297. 12873385 10.1016/s0896-6273(03)00428-8

[71] Mack V, Burnashev N, Kaiser KM, Rozov A, Jensen V, et al (2001) Conditional restoration of hippocampal synaptic potentiation in Glur-A-deficient mice. Science 292: 2501–2504. 11431570 10.1126/science.1059365

[72] Bannerman DM, Deacon RM, Brady S, Bruce A, Sprengel R, et al (2004) A comparison of GluR-A-deficient and wild-type mice on a test battery assessing sensorimotor, affective, and cognitive behaviors. Behav Neurosci 118: 643–647. 15174943 10.1037/0735-7044.118.3.643

[73] Wiedholz LM, Owens WA, Horton RE, Feyder M, Karlsson RM, et al (2008) Mice lacking the AMPA GluR1 receptor exhibit striatal hyperdopaminergia and ‘schizophrenia-related’ behaviors. Mol Psychiatry 13: 631–640. 17684498 10.1038/sj.mp.4002056

[74] Schmitt WB, Sprengel R, Mack V, Draft RW, Seeburg PH, et al (2005) Restoration of spatial working memory by genetic rescue of GluR-A-deficient mice. Nat Neurosci 8: 270–272. 15723058 10.1038/nn1412

[75] Reisel D, Bannerman DM, Schmitt WB, Deacon RM, Flint J, et al (2002) Spatial memory dissociations in mice lacking GluR1. Nat Neurosci 5: 868–873. 12195431 10.1038/nn910

[76] Zhou R, Holmes A, Du J, Malkesman O, Yuan P, et al (2009) Genome-wide gene expression profiling in GluR1 knockout mice: key role of the calcium signaling pathway in glutamatergically mediated hippocampal transmission. Eur J Neurosci 30: 2318–2326. 20092574 10.1111/j.1460-9568.2009.07022.x PMC3035979

[77] Li G, Hidaka H, Wollheim CB (1992) Inhibition of voltage-gated Ca2+ channels and insulin secretion in HIT cells by the Ca2+/calmodulin-dependent protein kinase II inhibitor KN-62: comparison with antagonists of calmodulin and L-type Ca2+ channels. Mol Pharmacol 42: 489–488. 1328847

[78] Ledoux J, Chartier D, Leblanc N (1999) Inhibitors of calmodulin-dependent protein kinase are nonspecific blockers of voltage-dependent K+ channels in vascular myocytes. J Pharmacol Exp Ther 290: 1165–1174. 10454491

[79] Tokumitsu H, Chijiwa T, Hagiwara M, Mizutani A, Terasawa M, et al (1990) KN-62, 1-[N,O-bis(5-isoquinolinesulfonyl)-N-methyl-L-tyrosyl]-4-phenylpiperazi ne, a specific inhibitor of Ca2+/calmodulin-dependent protein kinase II. J Biol Chem 265: 4315–4320. 2155222

[80] Sumi M, Kiuchi K, Ishikawa T, Ishii A, Hagiwara M, et al (1991) The newly synthesized selective Ca2+/calmodulin dependent protein kinase II inhibitor KN-93 reduces dopamine contents in PC12h cells. Biochem Biophys Res Commun 181: 968–975. 1662507 10.1016/0006-291x(91)92031-e

[81] Faas GC, Raghavachari S, Lisman JE, Mody I (2011) Calmodulin as a direct detector of Ca2+ signals. Nat Neurosci 14: 301–304. 21258328 10.1038/nn.2746 PMC3057387

[82] Feng B, Raghavachari S, Lisman J (2011) Quantitative estimates of the cytoplasmic, PSD, and NMDAR-bound pools of CaMKII in dendritic spines. Brain Res 1419: 46–52. 21925648 10.1016/j.brainres.2011.08.051 PMC3196057

[83] Chen HX, Otmakhov N, Strack S, Colbran RJ, Lisman JE (2001) Is persistent activity of calcium/calmodulin-dependent kinase required for the maintenance of LTP? J Neurophysiol 85: 1368–1376. 11287461 10.1152/jn.2001.85.4.1368

[84] Liao D, Jones A, Malinow R (1992) Direct measurement of quantal changes underlying long-term potentiation in CA1 hippocampus. Neuron 9: 1089–1097. 1334418 10.1016/0896-6273(92)90068-o

[85] Liao D, Hessler NA, Malinow R (1995) Activation of postsynaptically silent synapses during pairing-induced LTP in CA1 region of hippocampal slice. Nature 375: 400–404. 7760933 10.1038/375400a0

[86] Kerchner GA, Nicoll RA (2008) Silent synapses and the emergence of a postsynaptic mechanism for LTP. Nat Rev Neurosci 9: 813–825. 18854855 10.1038/nrn2501 PMC2819160

[87] Isaac JT, Nicoll RA, Malenka RC (1995) Evidence for silent synapses: implications for the expression of LTP. Neuron 15: 427–434. 7646894 10.1016/0896-6273(95)90046-2

[88] Durand GM, Kovalchuk Y, Konnerth A (1996) Long-term potentiation and functional synapse induction in developing hippocampus. Nature 381: 71–75. 8609991 10.1038/381071a0

[89] Wu G, Malinow R, Cline HT (1996) Maturation of a central glutamatergic synapse. Science 274: 972–976. 8875937 10.1126/science.274.5289.972

[90] Lisman J, Raghavachari S (2006) A unified model of the presynaptic and postsynaptic changes during LTP at CA1 synapses. Sci STKE 2006: re11. 17033044 10.1126/stke.3562006re11

[91] Raghavachari S, Lisman JE (2004) Properties of quantal transmission at CA1 synapses. J Neurophysiol 92: 2456–2467. 15115789 10.1152/jn.00258.2004

[92] Adesnik H, Nicoll RA, England PM (2005) Photoinactivation of native AMPA receptors reveals their real-time trafficking. Neuron 48: 977–985. 16364901 10.1016/j.neuron.2005.11.030

[93] Park M, Penick EC, Edwards JG, Kauer JA, Ehlers MD (2004) Recycling endosomes supply AMPA receptors for LTP. Science 305: 1972–1975. 15448273 10.1126/science.1102026

[94] Lledo PM, Hjelmstad GO, Mukherji S, Soderling TR, Malenka RC, et al (1995) Calcium/calmodulin-dependent kinase II and long-term potentiation enhance synaptic transmission by the same mechanism. Proc Natl Acad Sci U S A 92: 11175–11179. 7479960 10.1073/pnas.92.24.11175 PMC40594

[95] Pettit DL, Perlman S, Malinow R (1994) Potentiated transmission and prevention of further LTP by increased CaMKII activity in postsynaptic hippocampal slice neurons. Science 266: 1881–1885. 7997883 10.1126/science.7997883

[96] Shirke AM, Malinow R (1997) Mechanisms of potentiation by calcium-calmodulin kinase II of postsynaptic sensitivity in rat hippocampal CA1 neurons. J Neurophysiol 78: 2682–2692. 9356418 10.1152/jn.1997.78.5.2682

[97] Pi HJ, Otmakhov N, Lemelin D, De Koninck P, Lisman J (2010) Autonomous CaMKII can promote either long-term potentiation or long-term depression, depending on the state of T305/T306 phosphorylation. J Neurosci 30: 8704–8709. 20592192 10.1523/JNEUROSCI.0133-10.2010 PMC2903435

[98] Toni N, Buchs PA, Nikonenko I, Bron CR, Muller D (1999) LTP promotes formation of multiple spine synapses between a single axon terminal and a dendrite. Nature 402: 421–425. 10586883 10.1038/46574

[99] Jourdain P, Fukunaga K, Muller D (2003) Calcium/calmodulin-dependent protein kinase II contributes to activity-dependent filopodia growth and spine formation. J Neurosci 23: 10645–10649. 14627649 10.1523/JNEUROSCI.23-33-10645.2003 PMC6740921

[100] Ciani L, Boyle KA, Dickins E, Sahores M, Anane D, et al (2011) Wnt7a signaling promotes dendritic spine growth and synaptic strength through Ca(2)/Calmodulin-dependent protein kinase II. Proc Natl Acad Sci U S A 108: 10732–10737. 21670302 10.1073/pnas.1018132108 PMC3127879

[101] Williams SR, Mitchell SJ (2008) Direct measurement of somatic voltage clamp errors in central neurons. Nat Neurosci 11: 790–798. 18552844 10.1038/nn.2137

[102] Sugiura H, Yamauchi T (1994) Changes of the expression of protein substrates of Ca2+/calmodulin-dependent protein kinase II in neonate and adult rats. FEBS Lett 341: 299–302. 8137957 10.1016/0014-5793(94)80477-x

[103] Sugiura H, Yamauchi T (1994) Developmental changes of protein substrates of Ca2+/calmodulin-dependent protein kinase II in the rat forebrain. Brain Res 659: 42–54. 7820680 10.1016/0006-8993(94)90861-3

[104] Sugiura H, Yamauchi T (1992) Developmental changes in the levels of Ca2+/calmodulin-dependent protein kinase II alpha and beta proteins in soluble and particulate fractions of the rat brain. Brain Res 593: 97–104. 1333874 10.1016/0006-8993(92)91269-k

[105] Kelly PT, Shields S, Conway K, Yip R, Burgin K (1987) Developmental changes in calmodulin-kinase II activity at brain synaptic junctions: alterations in holoenzyme composition. J Neurochem 49: 1927–1940. 2824699 10.1111/j.1471-4159.1987.tb02456.x

[106] Ciani L, Boyle KA, Dickins E, Sahores M, Anane D, et al (2011) Wnt7a signaling promotes dendritic spine growth and synaptic strength through Ca(2)(+)/Calmodulin-dependent protein kinase II. Proc Natl Acad Sci U S A 108: 10732–10737. 21670302 10.1073/pnas.1018132108 PMC3127879

[107] Sharma G, Grybko M, Vijayaraghavan S (2008) Action potential-independent and nicotinic receptor-mediated concerted release of multiple quanta at hippocampal CA3-mossy fiber synapses. J Neurosci 28: 2563–2575. 18322100 10.1523/JNEUROSCI.5407-07.2008 PMC2696816

[108] Shen G, Van Sickle BJ, Tietz EI (2010) Calcium/calmodulin-dependent protein kinase II mediates hippocampal glutamatergic plasticity during benzodiazepine withdrawal. Neuropsychopharmacology 35: 1897–1909. 20445501 10.1038/npp.2010.61 PMC2904841

[109] Buard I, Coultrap SJ, Freund RK, Lee YS, Dell'Acqua ML, et al (2010) CaMKII “autonomy” is required for initiating but not for maintaining neuronal long-term information storage. J Neurosci 30: 8214–8220. 20554872 10.1523/JNEUROSCI.1469-10.2010 PMC2891520

[110] Goold CP, Nicoll RA (2010) Single-cell optogenetic excitation drives homeostatic synaptic depression. Neuron 68: 512–528. 21040851 10.1016/j.neuron.2010.09.020 PMC3111089

[111] Sanhueza M, Fernandez-Villalobos G, Stein IS, Kasumova G, Zhang P, et al (2011) Role of the CaMKII/NMDA receptor complex in the maintenance of synaptic strength. J Neurosci 31: 9170–9178. 21697368 10.1523/JNEUROSCI.1250-11.2011 PMC3138556

[112] Leonard AS, Lim IA, Hemsworth DE, Horne MC, Hell JW (1999) Calcium/calmodulin-dependent protein kinase II is associated with the N-methyl-D-aspartate receptor. Proc Natl Acad Sci U S A 96: 3239–3244. 10077668 10.1073/pnas.96.6.3239 PMC15926

[113] Barria A, Malinow R (2005) NMDA receptor subunit composition controls synaptic plasticity by regulating binding to CaMKII. Neuron 48: 289–301. 16242409 10.1016/j.neuron.2005.08.034

[114] Zhou Y, Takahashi E, Li W, Halt A, Wiltgen B, et al (2007) Interactions between the NR2B receptor and CaMKII modulate synaptic plasticity and spatial learning. J Neurosci 27: 13843–13853. 18077696 10.1523/JNEUROSCI.4486-07.2007 PMC6673634

[115] Hanley RM, Means AR, Ono T, Kemp BE, Burgin KE, et al (1987) Functional analysis of a complementary DNA for the 50-kilodalton subunit of calmodulin kinase II. Science 237: 293–297. 3037704 10.1126/science.3037704

[116] Rao A, Craig AM (1997) Activity regulates the synaptic localization of the NMDA receptor in hippocampal neurons. Neuron 19: 801–812. 9354327 10.1016/s0896-6273(00)80962-9

[117] Gomperts SN, Rao A, Craig AM, Malenka RC, Nicoll RA (1998) Postsynaptically silent synapses in single neuron cultures. Neuron 21: 1443–1451. 9883736 10.1016/s0896-6273(00)80662-5

[118] Petralia RS, Esteban JA, Wang YX, Partridge JG, Zhao HM, et al (1999) Selective acquisition of AMPA receptors over postnatal development suggests a molecular basis for silent synapses. Nat Neurosci 2: 31–36. 10195177 10.1038/4532

[119] Nusser Z, Lujan R, Laube G, Roberts JD, Molnar E, et al (1998) Cell type and pathway dependence of synaptic AMPA receptor number and variability in the hippocampus. Neuron 21: 545–559. 9768841 10.1016/s0896-6273(00)80565-6

[120] Takumi Y, Ramirez-Leon V, Laake P, Rinvik E, Ottersen OP (1999) Different modes of expression of AMPA and NMDA receptors in hippocampal synapses. Nat Neurosci 2: 618–624. 10409387 10.1038/10172

[121] Zhu JJ, Esteban JA, Hayashi Y, Malinow R (2000) Postnatal synaptic potentiation: delivery of GluR4-containing AMPA receptors by spontaneous activity. Nat Neurosci 3: 1098–1106. 11036266 10.1038/80614

[122] Esteban JA, Shi SH, Wilson C, Nuriya M, Huganir RL, et al (2003) PKA phosphorylation of AMPA receptor subunits controls synaptic trafficking underlying plasticity. Nat Neurosci 6: 136–143. 12536214 10.1038/nn997

[123] Man HY, Sekine-Aizawa Y, Huganir RL (2007) Regulation of {alpha}-amino-3-hydroxy-5-methyl-4-isoxazolepropionic acid receptor trafficking through PKA phosphorylation of the Glu receptor 1 subunit. Proc Natl Acad Sci U S A 104: 3579–3584. 17360685 10.1073/pnas.0611698104 PMC1805611

[124] Yasuda H, Barth AL, Stellwagen D, Malenka RC (2003) A developmental switch in the signaling cascades for LTP induction. Nat Neurosci 6: 15–16. 12469130 10.1038/nn985

[125] Chen M, Wan Y, Ade K, Ting J, Feng G, et al (2011) Sapap3 deletion anomalously activates short-term endocannabinoid-mediated synaptic plasticity. J Neurosci 31: 9563–9573. 21715621 10.1523/JNEUROSCI.1701-11.2011 PMC3367431

[126] Dosemeci A, Jaffe H (2010) Regulation of phosphorylation at the postsynaptic density during different activity states of Ca2+/calmodulin-dependent protein kinase II. Biochem Biophys Res Commun 391: 78–84. 19896464 10.1016/j.bbrc.2009.10.167 PMC2812614

[127] Ding J, Peterson JD, Surmeier DJ (2008) Corticostriatal and thalamostriatal synapses have distinctive properties. J Neurosci 28: 6483–6492. 18562619 10.1523/JNEUROSCI.0435-08.2008 PMC3461269

[128] Ding JB, Guzman JN, Peterson JD, Goldberg JA, Surmeier DJ (2010) Thalamic gating of corticostriatal signaling by cholinergic interneurons. Neuron 67: 294–307. 20670836 10.1016/j.neuron.2010.06.017 PMC4085694

[129] Smeal RM, Gaspar RC, Keefe KA, Wilcox KS (2007) A rat brain slice preparation for characterizing both thalamostriatal and corticostriatal afferents. J Neurosci Methods 159: 224–235. 16899300 10.1016/j.jneumeth.2006.07.007

[130] Roeper J, Lorra C, Pongs O (1997) Frequency-dependent inactivation of mammalian A-type K+ channel KV1.4 regulated by Ca2+/calmodulin-dependent protein kinase. J Neurosci 17: 3379–3391. 9133364 10.1523/JNEUROSCI.17-10-03379.1997 PMC6573714

[131] Park D, Coleman MJ, Hodge JJ, Budnik V, Griffith LC (2002) Regulation of neuronal excitability in Drosophila by constitutively active CaMKII. J Neurobiol 52: 24–42. 12115891 10.1002/neu.10066

[132] Nelson AB, Gittis AH, du Lac S (2005) Decreases in CaMKII activity trigger persistent potentiation of intrinsic excitability in spontaneously firing vestibular nucleus neurons. Neuron 46: 623–631. 15944130 10.1016/j.neuron.2005.04.009

[133] Cazorla M, Shegda M, Ramesh B, Harrison NL, Kellendonk C (2012) Striatal D2 receptors regulate dendritic morphology of medium spiny neurons via Kir2 channels. J Neurosci 32: 2398–2409. 22396414 10.1523/JNEUROSCI.6056-11.2012 PMC3564593

[134] Ashpole NM, Song W, Brustovetsky T, Engleman EA, Brustovetsky N, et al (2012) Calcium/calmodulin-dependent protein kinase II (CaMKII) inhibition induces neurotoxicity via dysregulation of glutamate/calcium signaling and hyperexcitability. J Biol Chem 10.1074/jbc.M111.323915 PMC3318689 22253441

