# Supplementary figures and images for: Genetic Inhibition of CaMKII in Dorsal Striatal Medium Spiny Neurons Reduces Functional Excitatory Synapses and Enhances Intrinsic Excitability
Source: PLoS One. 2012 Sep 21;7(9):e45323. doi: 10.1371/journal.pone.0045323 (PMC3448631; doi:10.1371/journal.pone.0045323)

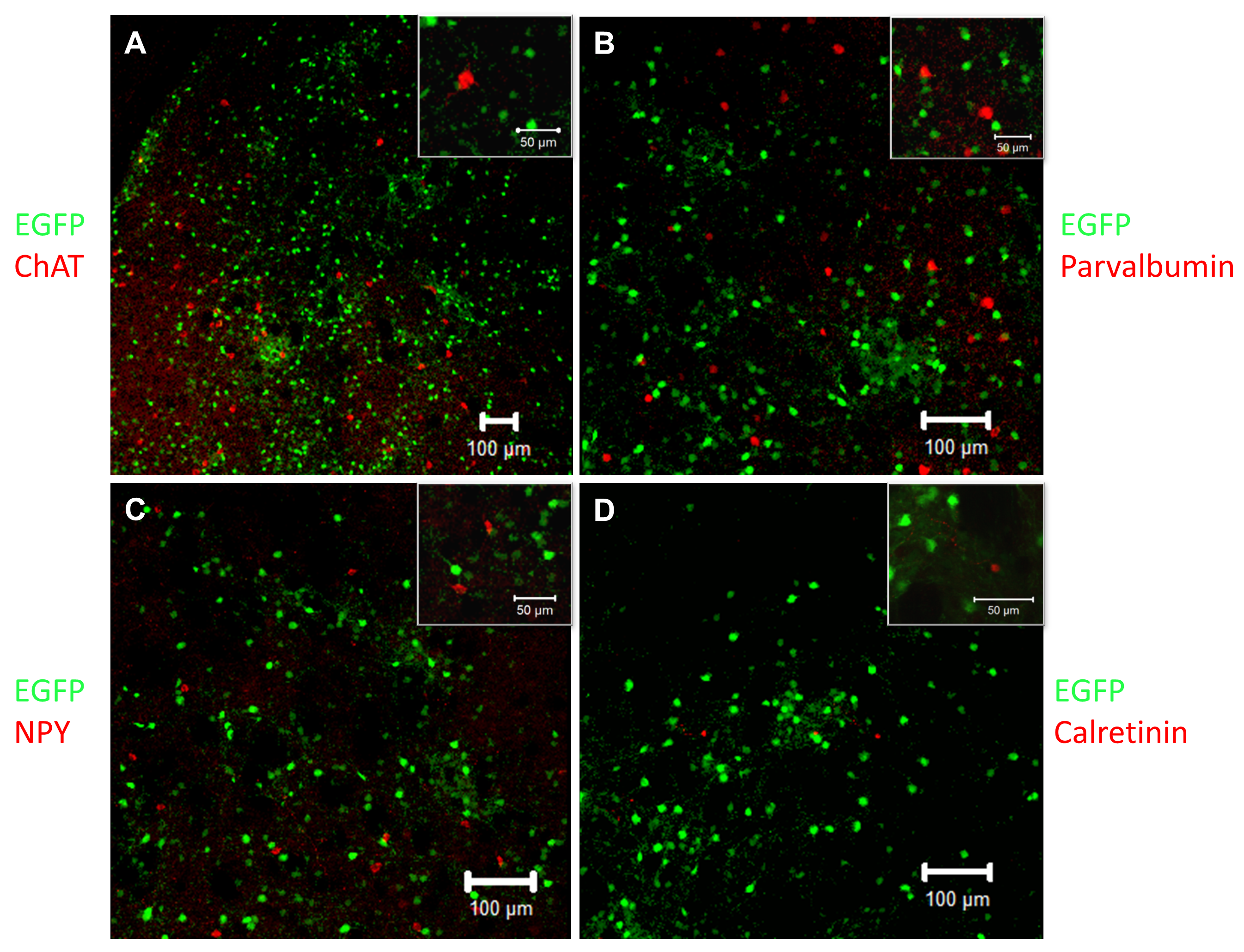

Supplement: Figure S1 — Striatal interneuron markers do not colocalize with EAC3I peptide. (A) 20× confocal image of dorsal lateral striatum from an EAC3I mouse of endogenous EGFP expression (green) and striatal ChAT immunopositive interneurons (red, 0/70 ChAT positive neurons contained EGFP). Scale bar 100 µm, inset 50 µm. (B) Like A, but striatal parvalbumin immunopositive interneurons labeled (red, 0/37 parvalbumin positive neurons contained EGFP). Scale bar 100 µm, inset 50 µm. (C) Like A, but striatal NPY immunopositive interneurons labeled (red, 0/22 NPY positive neurons contained EGFP). Scale bar 100 µm, inset 50 µm. (D) Like A, but striatal calretinin immunopositive interneurons labeled (red, 0/15 calretinin positive neurons contained EGFP). Scale bar 100 µm, inset 50 µm. (TIF) [file pone.0045323.s001.tif]
